# Stimmungsbild des ärztlichen Nachwuchses in der Frauenheilkunde und Geburtshilfe in Norddeutschland: Eine umfassende Bestandsaufnahme ausbildender Kliniken im Bereich der Norddeutschen Gesellschaft für Gynäkologie und Geburtshilfe mit Charakterisierung des ärztlichen Nachwuchses und einer praxisorientierten Herangehensweise in Vorbereitung auf die Generation Z

**DOI:** 10.1007/s00129-022-04942-5

**Published:** 2022-05-02

**Authors:** Jann Lennard Scharf, Arne Bringewatt, Christoph Dracopoulos, Achim Rody, Michael Gembicki

**Affiliations:** 1 Klinik für Frauenheilkunde und Geburtshilfe, Universitätsklinikum Schleswig-Holstein, Campus Lübeck, Ratzeburger Allee 160, 23538 Lübeck, Deutschland; 2 Frauenklinik, Westküstenklinikum, Esmarchstr. 50, 25746 Heide, Deutschland

**Keywords:** Querschnittsstudien, Selbstfürsorge, Arbeitsbelastung, Umfragen und Fragebögen, Weiterbildung, Cross-sectional studies, Self-care, Workload, Surveys and questionnaires, Education, continuing

## Abstract

**Hintergrund:**

Der ärztliche Nachwuchs hat disruptive Effekte und macht auch vor dem Fach Frauenheilkunde und Geburtshilfe nicht halt. Noch fokussiert sich der Diskurs auf die Generation Y (1980–1994). Um dem Nachwuchs ein konstruktives Arbeitsumfeld zu bieten, drängt die Zeit. Es gilt, sich dessen Anforderungen an ein solches zu vergegenwärtigen.

**Zielsetzung:**

Erfassen des Stimmungsbilds des ärztlichen Nachwuchses in der Frauenheilkunde und Geburtshilfe mit anschließender Ableitung praxisrelevanter Aspekte unter Berücksichtigung der künftig dominierenden Generation Z (1995–2009).

**Methoden:**

Von Januar bis Oktober 2021 wurde eine deskriptive Querschnittserhebung des ärztlichen Nachwuchses ausbildender Kliniken im Fach Frauenheilkunde und Geburtshilfe durchgeführt. Es wurden 81 Fragen zu 6 Themen online abgefragt.

**Ergebnis:**

Ausgewertet wurden 122 Fragebögen (*n* = 122): 28 % (*n* = 33) schätzen die Arbeitsbelastung als sehr hoch, 56 % (*n* = 67) als hoch ein. Zwei Drittel (*n* = 81) arbeiten wöchentlich 40–59 h. Den Anteil delegierbarer Tätigkeiten beziffern 67 % (*n* = 80) auf > 25 %. 88 % (*n* = 105) verbringen 25–75 % der täglichen Arbeitszeit mit Dokumentieren. 92 % (*n* = 109) wünschen sich regelmäßige Ober- bzw. Chefarztvisiten, 81 % (*n* = 95) beurteilen die Weiterbildung schlechter als gut. Für 32 % (*n* = 38) besteht ein ausgeglichenes Verhältnis zwischen Gesundheit und Beruf, 25 % (*n* = 29) beurteilen die Arbeitsbedingungen als familienfreundlich, und 88 % (*n* = 102) wären bereit, bei anhaltender Unzufriedenheit den Arbeitgeber zu wechseln.

**Schlussfolgerung:**

Den Nachwuchs dominieren Forderungen nach Weiterbildung, Teilzeit, Sinnhaftigkeit, Vereinbarkeit von Familie und Beruf, Wertschätzung und Selbstfürsorge. Lösungskonzepte, um diesen gerecht zu werden, stünden zur Verfügung.

**Zusatzmaterial online:**

Zusätzliche Informationen sind in der Online-Version dieses Artikels (10.1007/s00129-022-04942-5) enthalten.

## Abstract (en)

**Background:**

The new generation of physicians has disruptive effects and also does not stop at the discipline of obstetrics and gynecology. The discourse is still focused on Generation Y (1980–1994). In order to offer a constructive working environment to the new generation, time is pressing. It is important to be aware of their requirements for such an environment.

**Objective:**

Determination of the pattern of opinions of the next generation of physicians in gynecology and obstetrics and then to derive aspects relevant to the practice, taking the future dominant Generation Z (1995–2009) into account.

**Methods:**

A descriptive cross-sectional survey of junior physicians in gynecology and obstetrics at training hospitals was conducted from January to October 2021. A total of 81 questions on 6 topics were answered online.

**Results:**

A total of 122 questionnaires (*n* = 122) were evaluated. Of these 28% (*n* = 33) rated the workload as very high and 56% (*n* = 67) as high. Two thirds (*n* = 81) worked 40–59 h per week, 67% (*n* = 80) put the share of delegable activities at > 25%, 88% (*n* = 105) spent 25–75% of daily work time on documentation, 92% (*n* = 109) would like to have regular senior or chief physician visits. Of the participants 81% (*n* = 95) rated continuing education as less than good. For 32% (*n* = 38) there was a balanced relation between health and profession, 25% (*n* = 29) rated working conditions as family friendly and 88% (*n* = 102) would be willing to change employers if dissatisfaction persisted.

**Conclusion:**

The next generation of physicians is dominated by demands for further training, part-time work, a sense of purpose, compatibility of family and career, appreciation and self-care. Solution concepts to meet these demands would be available.

## Einleitung

Das Interesse an derzeit den Nachwuchs repräsentierenden Generationen ist weiterhin ungebrochen, ergibt zumindest eine Abfrage über den Online-Dienst *Google Trends*. Gleichermaßen erfährt eine Auseinandersetzung mit dem ärztlichen Nachwuchs in der wissenschaftlichen Literatur zunehmende Beachtung [1]. Um an dem Diskurs über den zukünftigen ärztlichen Nachwuchs zu partizipieren, ist es unabdingbar, sich bereits vorab die Anforderungen Medizinstudierender zu vergegenwärtigen. Nach einer bundesweiten Onlinebefragung aller medizinischen Fakultäten an 9079 Medizinstudierenden werteten Kasch et al. 21 Fragen zur zukünftigen Arbeitsplatzwahl und zur erwarteten Arbeitsplatzzufriedenheit aus und beschrieben 5 die Erwartungshaltung charakterisierende Faktoren [2].

Eine Auseinandersetzung mit dem ärztlichen Nachwuchs wird in der Literatur zunehmend beachtet

Darüber hinaus nimmt die Berücksichtigung unterschiedlicher Generationencluster im Gesundheitswesen an Fahrt auf [3–6], und die Sensibilisierung gegenüber den Anforderungen und Bedürfnissen der jungen Ärztegeneration rückt fächerübergreifend in den Vordergrund [7–11]. Zunehmend erreicht die Auseinandersetzung mit der nachfolgenden Generation auch das Fachgebiet Frauenheilkunde und Geburtshilfe [10, 12–15]. Dabei bedarf es stetiger Anstrengungen, um die Generationencluster in diesem Fachgebiet weiterhin zu charakterisieren, Arbeitsbedingungen und -belastung inklusive deren Auswirkungen auf den ärztlichen Nachwuchs zu eruieren, Weiterbildung und Nachwuchsförderung zu optimieren und praxistaugliche Lösungen in den Klinikalltag zu implementieren.

## Zielsetzung

Im Hinblick auf die Notwendigkeit einer umfassenden Auseinandersetzung mit dem ärztlichen Nachwuchs trägt diese Arbeit dazu bei, dessen Stimmung in der Frauenheilkunde und Geburtshilfe zu erfassen, dem wachsenden Interesse an der Generation Z gerecht zu werden, gleichzeitig für die junge Generation zu sensibilisieren und zum aktiven Mitgestalten zu ermutigen.

## Methoden

Zwischen Januar und Oktober 2021 wurde eine deskriptive Querschnittstudie unter Verwendung einer selbsterarbeiteten Umfrage online an Ärztinnen und Ärzten in Weiterbildung zur Fachärztin bzw. zum Facharzt für Frauenheilkunde und Geburtshilfe bzw. Fachärztinnen und -ärzten mit Anstellung an einer Klinik im Bereich der Norddeutschen Gesellschaft für Gynäkologie und Geburtshilfe (NGGG^1^) durchgeführt. Mit Hilfe des deutschen Krankenhausverzeichnisses^2^ wurden die Kliniken mit einer Abteilung für Frauenheilkunde und/oder Geburtshilfe im Einzugsgebiet der NGGG kontaktiert. Unterstützend wurden die jeweiligen Berufsverbände sowie die Onlinefortbildung *Gyn To Go*^3^ angeschrieben, um das Umfrageangebot zu verbreiten. Der Fragebogen umfasst 81 Fragen und gliedert sich in 6 Abschnitte: 1. Allgemeines, 2. Arbeitsbedingungen und -belastung, 3. Weiterbildung, 4. Freie Zeit, Gesundheit und Familie, 5. Rekrutierung und Bindung sowie 6. Sonstiges. Für eine gültige Wertung sollten die Fragen des Abschnitts *Allgemeines* beantwortet worden sein. Analog zu Mitchell ordneten wir die Jahrgänge 1980 bis 1994 der Generation Y und die der Jahrgänge 1995 bis 2009 der Generation Z zu [11]. Je nach Fragestellung erfolgten die Beantwortungen per Nominal‑, Ordinal- oder Intervallskala sowie darüber hinaus per diverser Auswahlmöglichkeiten oder als Freitext. Darstellung und anonymisierte Auswertung erfolgten mit dem Softwaretool SurveyMonkey®^4^. Mehrfachteilnahmen wurden mittels IP(Internet-Protokoll)-Adressprüfung ausgeschlossen, waren aber letztendlich von den teilnehmenden Personen unter Einhaltung der Teilnahmebedingungen abhängig. Aus Gründen der Übersicht wurde durchweg das generische Maskulinum verwendet. Die Auswertung erfolgt für jede einzelne Frage. Die komplette Auswertung der Umfrage ist dem Anhang zu entnehmen (s. Zusatzmaterial online).

## Ergebnisse

Insgesamt wurden 141 Fragebögen erfasst, von denen die meisten (*n* = 122) ausgewertet werden konnten. 19 Fragebögen wurden unzureichend beantwortet. Die charakterisierenden Merkmale der Teilnehmenden zeigt Tab. 1.

**Table 1:** 

Frage	Antworten	*n* (%)
Geburtsjahrgang	1965–1979	11 (9)
	1980–1994	105 (86)
	1995–2009	6 (5)
Geschlecht	Männlich	25 (20)
	Weiblich	96 (79)
	Divers	1 (1)
Weiterbildungsjahr	1	15 (12)
	2	12 (10)
	3	21 (17)
	4	16 (13)
	5	19 (16)
	> 6	6 (5)
	Facharzt	33 (27)
Promotion	Ja	64 (52)
	Nein	30 (25)
	In Arbeit	28 (23)
Gründung einer Familie	Ja	47 (39)
	Nein	75 (61)
Vollzeit oder Teilzeit	Vollzeit	96 (79)
	Teilzeit	26 (21)
Arbeitgeber	Universitätsklinikum	45 (37)
	Akademisches Lehrkrankenhaus	55 (45)
	Kommunale Klinik	22 (18)

### Arbeitsbedingungen und -belastung

**Figure 1:**
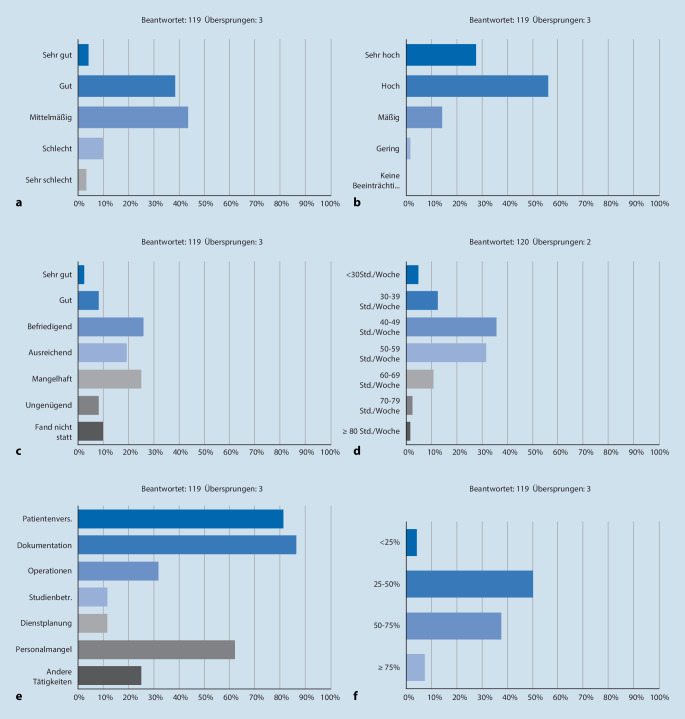

39 % (*n* = 46) beurteilen ihre Arbeitsbedingungen als gut, 44 % (*n* = 52) als mittelmäßig (Abb. 1a), 28 % (*n* = 33) schätzen ihre Arbeitsbelastung als sehr hoch, 56 % (*n* = 67) als hoch ein (Abb. 1b). 63 % (*n* = 75) beurteilen ihre Einarbeitung zu Berufsstart bzw. zu Beginn einer neuen Rotation höchstens als ausreichend (Abb. 1c). 68 % (*n* = 81) gaben an, mit ihrem Einkommen zufrieden zu sein, 33 % (*n* = 39), es in Bezug auf ihre geleistete Arbeit und Verantwortung für angemessen zu halten. Zwei Drittel der Befragten (*n* = 81) arbeiten wöchentlich 40–59 h (Abb. 1d), 53 % (*n* = 63) präferieren eine durchschnittliche Wochenarbeitszeit von 30–39 h, weitere 32 % (*n* = 38) 40–49 h. 63 % (*n* = 75) bevorzugen eine flexible Arbeitszeit. 90 % (*n* = 108) halten ihre gesetzlich vorgeschriebenen Arbeitspausen nicht ein, und wenn, verlassen 58 % (*n* = 7) dabei den Arbeitsplatz nicht. 91 % (*n* = 108) leisten regelmäßig Überstunden, wobei die durchschnittlichen wöchentlichen Überstunden zu 84 % (*n* = 100) zwischen einer und 10 h betragen. Diese entstehen in 87 % (*n* = 103) durch nicht in der regulären Arbeitszeit erledigte Dokumentation, in 82 % (*n* = 97) durch die Patientinnenversorgung. Personalmangel geben 62 % (*n* = 74) als Überstundengrund an (Abb. 1e). Der Anteil delegierbarer, nichtärztlicher Tätigkeiten wird von 33 % (*n* = 39) auf < 25 %, von 52 % (*n* = 62) auf 25–50 % beziffert. Die Hälfte der Befragten (*n* = 60) verbringt 25–50 % ihrer täglichen Arbeitszeit mit Dokumentieren, weitere 38 % (*n* = 45) 50–75 % dieser Zeit (Abb. 1f). 44 % (*n* = 52) sind zufrieden mit dem Führungsstil ihres Chefarztes, für 21 % (*n* = 25) hat der Chefarzt und für 30 % (*n* = 36) haben die Oberärzte eine Vorbildfunktion. 92 % (*n* = 109) wünschen sich regelmäßige Oberarzt- bzw. Chefarztvisiten. Zu 62 % (*n* = 74) finden regelmäßige Chefarzt-, zu 53 % (*n* = 65) Oberarztvisiten statt. 47 % (*n* = 56) fühlen sich in ihrer Arbeit wertgeschätzt, und 53 % (*n* = 63) sind zufrieden mit ihrer beruflichen Situation. Bei Unzufriedenheit werden als die 3 häufigsten Gründe mit 77 % (*n* = 43) mangelnde Weiterbildung, mit 73 % (*n* = 41) fehlende Wertschätzung der eigenen Arbeit und mit 71 % (*n* = 40) zu hoher Dokumentationsaufwand genannt. Die kollegiale Zusammenarbeit bewerten 30 % (*n* = 35) als sehr gut, 50 % (*n* = 59) als gut, für 67 % (*n* = 79) leidet sie unter der Arbeitsbelastung.

### Weiterbildung

**Figure 2:**
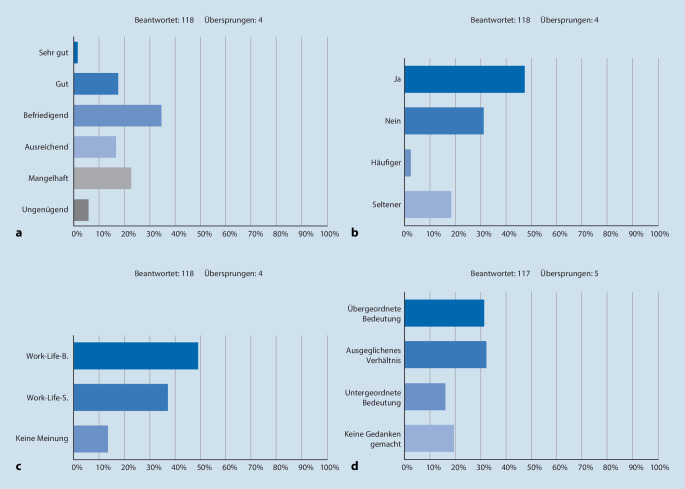

81 % (*n* = 95) beurteilen die Weiterbildung an ihrer Klinik schlechter als gut (Abb. 2a). Ein strukturierter Weiterbildungsplan fehlt in 81 % (*n* = 96). 74 % (*n* = 87) sind der Meinung, dass sie die Fähigkeiten, die sie während ihrer bisher absolvierten Weiterbildungszeit erlernt haben, in gleicher Weise effektiver hätten erlernen können. Ein Oberarzt-Mentoren-Programm ist in 27 % (*n* = 32) etabliert. Weiterbildungsgespräche finden für 47 % der Befragten (*n* = 56) jährlich statt und werden von 40 % (*n* = 46) als konstruktiv empfunden (Abb. 2b).

### Freie Zeit, Gesundheit und Familie

49 % (*n* = 58) bevorzugen eine ausgewogene *Work-Life-Balance* (Abb. 2c). Für 32 % (*n* = 37) nimmt die eigene Gesundheit einen übergeordneten Stellenwert ein, für 32 % (*n* = 38) besteht ein ausgeglichenes Verhältnis zwischen eigener Gesundheit und Beruf, und 16 % (*n* = 19) ordnen diese dem Beruf unter (Abb. 2d). 28 % (*n* = 32) benannten ihre Belastung am Arbeitsplatz ursächlich für eine Krankmeldung. 81 % (*n* = 95) präferieren mehr freie Zeit gegenüber mehr Einkommen, 8 % (*n* = 9) bevorzugen die Auszahlung von Überstunden, 50 % (*n* = 59) die Vergütung als Freizeitausgleich, 41 % (*n* = 48) eine Kombination aus beidem. 65 % (*n* = 76) sehen ihr Privatleben durch ihre Arbeitsbelastung negativ beeinträchtigt. 25 % (*n* = 29) beurteilen die Arbeitsbedingungen an ihrer Klinik als familienfreundlich. Ein Wechsel in die Teilzeit stellt für 69 % (*n* = 82) unabhängig vom Sozialleben eine Option dar.

### Rekrutierung und Bindung

**Figure 3:**
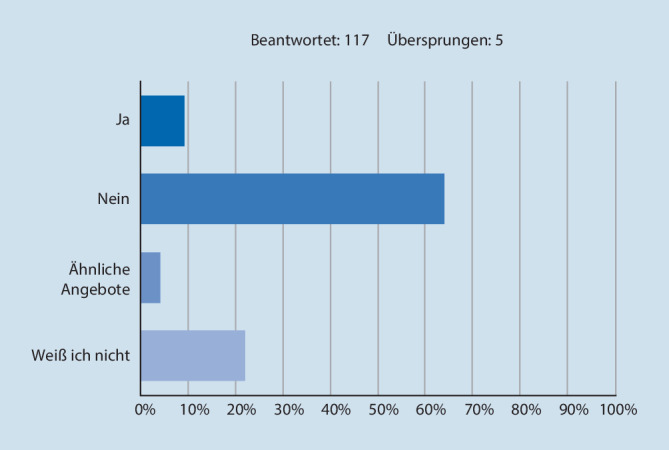

21 % (*n* = 24) ist die DGGG(Deutsche Gesellschaft für Gynäkologie und Geburtshilfe)-Nachwuchskampagne *GYN-WERDEN* geläufig. 64 % (*n* = 75) der Kliniken bieten keine Angebote an, um Nachwuchs zu rekrutieren (Abb. 3), 26 % (*n* = 30) geben an, ihre Klinik fördere Angebote, um sie im Arbeitsverhältnis zu halten. Als die 3 häufigsten Beweggründe, sich an einer Klinik zu bewerben, werden mit 47 % (*n* = 55) Wohnortnähe, mit 39 % (*n* = 45) das Operations- und Leistungsspektrum und mit 38 % (*n* = 44) die Geburtenzahl angegeben. 88 % (*n* = 102) wären bereit, bei anhaltender Unzufriedenheit den Arbeitgeber zu wechseln.

### Sonstiges

In 66 % (*n* = 77) kodieren Ärzte ohne adäquate Kenntnisse Diagnosen und Prozeduren. 44 % (*n* = 51) wussten bei Einstellung, was die *Opt-Out-*Regelung beinhaltet. 51 % (*n* = 59) unterschrieben diese. In 28 % (*n* = 33) war die Unterzeichnung der Regelung Voraussetzung für die Einstellung. In 21 % (*n* = 24) bietet die Klinik eine Supervision zur Verarbeitung belastender Erlebnisse an.

## Diskussion

Über *Google Trends* lässt sich im Verlauf der letzten 10 Jahre eine exponentielle Zunahme der Generation-Z-bezogenen Suchanfragen aufzeigen. Zeitgleich sank zumindest in Bezug auf die Suchanfragen das Interesse an der Generation Y. Schon Shatto et al. resümierten 2016 folgerichtig: „Much has been written about teaching Millennials; however, little has been discussed about Generation Z“ [16]. Bereits 2008 wurde die voraussichtliche Einstellung von nach 1995 geborenen Ärztinnen und Ärzten ab dem Jahr 2017 erwartet [11]. Somit bestand ausreichend Zeit, sich mit dieser Generation auseinanderzusetzen und auf das gesteigerte Interesse zu reagieren. Der Anteil dieser Generation an der deutschen Erwerbsbevölkerung im Jahr 2020 wird auf 9,5 % geschätzt [17] – in akademisierten Berufen dürfte dieser deutlich niedriger ausfallen. Im Gesundheitswesen und deren akademisierten Spezialisierungen müssen wir uns derzeit damit begnügen, durch Erhebung des gegenwärtigen Zustandes überwiegend anhand der Generation Y Erkenntnisse auf deren Nachfolger zu antizipieren. Diese Annahme übertragen wir auf die Frauenheilkunde und Geburtshilfe, einem Fachgebiet, in dem die Mehrheit der Beschäftigten weiblich ist (Tab. 1; [18]). Schmidt et al. erarbeiteten Lösungsvorschläge für die Anforderungen der Generation Y an den Arbeitsplatz Krankenhaus. Hierzu zählen Maßnahmen für Rekrutierung, Motivation, Ausbildung, Entwicklung und Bindung von Personal [10]. Sie können als Orientierungshilfe herangezogen werden. Die meisten Maßnahmen erzielen große Wirkung bei geringem Aufwand. Für den Fachbereich Frauenheilkunde und Geburtshilfe besteht hier immenses Optimierungspotenzial.

Für den Fachbereich Frauenheilkunde und Geburtshilfe besteht immenses Optimierungspotenzial

Erhebungen der Arbeitsbedingungen und -belastungen angestellter Ärztinnen und Ärzte werden regelmäßig im *MB(Marburger Bund)-Monitor* veröffentlicht [8, 19]. Unsere Rückmeldungen stützen den zuletzt 2019 erhobenen Trend, dass die Arbeitsrahmenbedingungen als unzureichend und die Arbeit selbst als übermäßige Belastung empfunden werden. Auch die wöchentliche Arbeitszeit verbleibt hoch, wie bereits 2015 durch das *junge Forum* festgestellt wurde [12, 19]. Insbesondere bei der tatsächlichen Wochenstundenzahl wünschen sich die Kolleginnen und Kollegen eine Veränderung. Bereits jetzt besteht eine hohe Nachfrage an Teilzeitstellen [8] – für die Mehrheit der befragten Ärztinnen und Ärzte eine attraktive Alternative. Immerhin geben 70 % den Wunsch nach Reduktion der Wochenarbeitszeit an. Sogar Vergütungseinbußen scheinen zugunsten der körperlichen und seelischen Gesundheit sowie einem ausgeglichenen Sozialleben akzeptiert zu werden. Noch arbeiten zwei Drittel der Befragten in Vollzeit. Unter Berücksichtigung des Teilnehmendenalters und des Frauenanteils sollten die Arbeitgeber diese Sichtweise ernst nehmen [10]. Eine hohe Wochenarbeitszeit, Überstunden, die *Work-Life-Imbalance*, die Diskrepanz zwischen Einkommen und Verantwortung, die negativen Auswirkungen der Arbeit auf das Privatleben sowie ein zunehmendes Gesundheitsbewusstsein nähren einen *Circulus vitiosus*. Dies treibt den Nachwuchs in die Teilzeit, zumal mehr Arbeit bei gleichzeitig mehr Einkommen keinen Anreiz bietet. An dieser fatalen Entwicklung können Arbeitgeber kein Interesse haben. Die anfallenden durchschnittlichen wöchentlichen Überstunden bergen möglicherweise weniger Konfliktpotenzial als vielmehr deren Ursachen: Der während der regulären Arbeitszeit nicht zu bewältigende Wust an Dokumentation kombiniert mit dem hohen Anteil delegierbarer, nichtärztlicher Tätigkeiten bei gleichzeitigem Personalmangel und zu gewährleistender Patientinnenversorgung wird gerade die Generation Z nicht hinnehmen, da diese besonders viel Wert auf sinnhafte Arbeit legt [14, 17, 20]. Dies ist gleichermaßen auf das Kodieren von Diagnosen und Prozeduren ohne adäquate Kenntnisse übertragbar. Der Führungsstil einer Chefärztin bzw. eines Chefarztes ist bedeutender denn je, um angesichts der mittlerweile stark voneinander divergierenden Anforderungen den einzelnen Generationenclustern innerhalb einer Klinik gerecht zu werden. Vorreiter auf dem Gebiet der Mitarbeiterführung waren Bass und Avolio, die das *Full Range of Leadership* entwickelten [21]. Dieses Führungsmodell soll das gesamte Spektrum des Führungsverhaltens abbilden [3, 5, 22, 23]. Seine Kenntnis kann für Führungskräfte angesichts der Umfragewerte im Klinikalltag hilfreich sein. Es gilt darüber hinaus, die Stärken der Mitarbeiterinnen und Mitarbeiter zu fördern, was zuletzt während der COVID-19-Pandemie vernachlässigt wurde, um die emotionale Mitarbeiterbindung aufrechtzuerhalten. Gerade Personal mit einer hohen emotionalen Mitarbeiterbindung ist engagiert, loyal und produktiv und weist wenige Fehltage sowie eine geringe Fluktuation auf [24]. Die Wertschätzung der als Ärztin oder Arzt in Weiterbildung erbrachten Arbeit hat enorm an Bedeutung gewonnen, wird in der Realität jedoch nicht ausreichend gewürdigt [10, 17]. Der Wunsch nach kontinuierlichen Chef- oder Oberarztvisiten ist unverkennbar, ermöglichen diese einen generationenübergreifenden Austausch, gewährleisten als *Bedside-Teaching* eine Supervision, fördern die Weiterbildung und stellen dadurch die Versorgung der Patientinnen und Patienten sicher.

Bei anhaltender Unzufriedenheit scheut der Nachwuchs keinesfalls davor zurück, den Arbeitgeber zu wechseln. Umso verwunderlicher ist, dass Kliniken Angebote nicht ausschöpfen, um Personal zu rekrutieren, auszubilden und zu binden.

Zu fördern sind die Stärken von Mitarbeitenden auch im Hinblick auf die emotionale Bindung

Die Disruption der Lernkultur im Fach der Frauenheilkunde und Geburtshilfe ist anerkannt [25–28]. Der umfangreiche Weiterbildungskatalog sowie die Spezialisierungsmöglichkeiten zeichnen die Frauenheilkunde und Geburtshilfe aus [29]. Warum wird dieser Trumpf nicht ausgespielt? Die Weiterbildungsqualität ist ausbaufähig [13]. Ein strukturierter Weiterbildungsplan – bei Weitem keine Selbstverständlichkeit [30] – trägt zur Effektivität der fachärztlichen Ausbildung bei, an der es derzeit mangelt [10]. Bereits zu Berufsstart oder zu Beginn einer neuen Rotation fehlen effiziente Einarbeitungskonzepte. Dabei kann ein Oberarzt-Mentoren-Programm die Weiterbildung positiv verstärken [10, 17]. Auch Weiterbildungsgespräche erzielen positiv verstärkende Effekte und wirken nachhaltig [10, 17]. Diese finden zwar, wie gefordert, einmal jährlich und seltener häufiger statt, lassen aber konstruktive Kritik vermissen. Die Generation Z wünscht sich kontinuierliche Rückmeldungen, zumindest einmal alle 3 Monate. Das bisher etablierte Jahresgespräch verkommt zum Auslaufmodell [17].

Eine *Work-Life-Balance* mit fließendem Übergang zwischen Privat- und Berufsleben, geprägt durch flexible Arbeitszeiten, wird generell von der Generation Y bevorzugt, wohingegen eine *Work-Life-Separation* mit klarer Trennung zwischen Privat- und Berufsleben die „Zler“ ansprechen soll. Diese Annahme kann, unter Berücksichtigung des geringen Anteils der zwischen 1995 und 2009 Geborenen innerhalb der Studienpopulation, anhand dieser Umfrage nicht bestätigt werden. Scholler et al. propagieren, dass diese *Separation* ein Mythos sei, was einmal mehr die Heterogenität der Generationencluster betont [17, 20, 26]. Bereits während des Medizinstudiums, der entscheidenden Phase der Aneignung des *ärztlichen Habitus*, der Einstellung zur eigenen Gesundheit und Krankheit, werden angehende Mediziner gegenüber den gesundheitlichen Risiken des späteren ärztlichen Berufslebens und der Bedeutung der Selbstfürsorge sensibilisiert [31–35]. An dieser Stelle seien diverse Initiativen der Universität zu Lübeck für die Förderung des Studierens bei guter psychosozialer Gesundheit erwähnt [32]. Im Angesicht dieses Gesinnungswechsels wurde 2017 der Begriff *Wohlbefinden* zur Genfer Deklaration des Weltärztebundes hinzugefügt [36–39]. Auch das Beschlussprotokoll des 122. Deutschen Ärztetages formuliert diesbezüglich klare Forderungen [31, 39, 40]. Es gilt heute mehr denn je, den *ärztlichen Habitus* zu überwinden. Derzeit spiegelt sich die Vernachlässigung der Selbstfürsorge unter anderem in der Missachtung der gesetzlichen Arbeitspausen, der akzeptierten Ausweitung des Arbeitszeitgesetzes im Rahmen der *Opt-Out-*Regelung, der ausufernden Bürokratie oder der hohen Arbeitsbelastung wider. Zunehmend rückt der Erhalt der ärztlichen Gesundheit in den Fokus, dennoch bedarf es weiterer Anstrengungen [40]. Die Generation Z hat den Stellenwert der eigenen Gesundheit sowie des eigenen Wohlbefindens und deren Schlüsselfunktion als Indikatoren der Qualität der Versorgung von Patientinnen und Patienten längst erkannt [11, 32, 41].

Zunehmend rückt der Erhalt der ärztlichen Gesundheit in den Fokus

Nachwuchsrekrutierungsprogramme wie die *DGGG-Summer-School*^5^ bleiben trotz des offensichtlichen Erfolges eine Rarität [42, 43]. Selbst die aktuelle DGGG-Nachwuchskampagne* GYN-WERDEN*^6^ ist nur wenigen ein Begriff. Die Nachwuchsrekrutierung über das praktische Jahr scheinen viele Kliniken nicht auszuschöpfen. Immerhin gilt es als erwiesen, dass bereits die Famulatur die späteren Berufsentscheidungen nachhaltig beeinflusst [44].

### Limitation

Die auf ein geographisches Gebiet beschränkte Umfrage mit geringer Teilnehmendenzahl kann die Repräsentativität der Studienpopulation beeinträchtigen, zumal derzeit in akademisierten Berufen erst wenige „Zler“ beschäftigt sind. Es blieb daher bei einer rein deskriptiven Auswertung. Die Anzahl der für diese Erhebung kontaktierten Ärztinnen und Ärzte und damit auch die Rücklaufquote sind unbekannt. Streng genommen hängt die Teilnahme an dieser Umfrage von der Einhaltung der Teilnahmebedingungen ab. Durch die Verbreitung der Umfrage über *Gyn To Go* wurden sicherlich auch Ärztinnen und Ärzte außerhalb Norddeutschlands erreicht, obwohl diese nicht den Einschlusskriterien entsprachen. Die Beantwortungen könnten durch teils nichtvalidierte, möglicherweise suggestive Fragen beeinflusst und durch einerseits motivierte, andererseits frustrierte Ärztinnen und Ärzte entsprechend verzerrt worden sein. Das durch *Lockdowns* geprägte Pandemiejahr 2021 kann sich ebenfalls auf die Beantwortungen ausgewirkt haben.

## Fazit für die Praxis

- Die Generation Y hat die längste Zeit den ärztlichen Nachwuchs repräsentiert. Der Übergang zur Generation Z hat begonnen.
- Demographischer Wandel, Fachkräftemangel, zunehmende Ökonomisierung und Arbeitsverdichtung sorgen weiterhin für verbesserungswürdige Arbeitsbedingungen.
- Das Streben nach einem ausgeglichenen Sozialleben überwiegt.
- Es besteht eine hohe Arbeitsbelastung junger Ärztinnen und Ärzte in der Frauenheilkunde und Geburtshilfe.
- All dies wirkt sich nachweislich negativ aus auf ihre Weiterbildung, ihre Gesundheit und ihr Privatleben sowie auf die Nachwuchsförderung.
- Gleichzeitig dominieren Forderungen nach Teilzeit, Sinnhaftigkeit, Vereinbarkeit von Familie und Beruf, Wertschätzung und Selbstfürsorge den Nachwuchs. An konstruktiven Lösungskonzepten mangelt es nicht. Ausbildende Kliniken müssen sich diesen Herausforderungen stellen.

## Supplementary Information





## Footnotes

1 Norddeutsche Gesellschaft für Gynäkologie und Geburtshilfe (NGGG): http://www.nggg-gyn.de/ [Stand: 05.02.2022].

2 Deutsches Krankenhausverzeichnis: https://www.deutsches-krankenhaus-verzeichnis.de/app/suche [Stand: 05.02.2022].

3 *Gyn To Go*: https://gyn.medizintogo.de/ [Stand: 05.02.2022].

4 *SurveyMonkey*®: https://www.surveymonkey.de/ [Stand: 05.02.2022].

5 *DGGG-Summer-School*: https://www.dggg.de/weiterbildung-nachwuchs/junges-forum/summer-school [Stand: 05.02.2022].

6 GYN-WERDEN: https://www.gyn-werden.de/ [Stand: 05.02.2022].
