# Supplementary material for: Pattern of opinions of young physicians in gynecology and obstetrics in northern Germany: A comprehensive survey of training clinics in the area of the North German Society for Gynecology and Obstetrics with characterization of the next generation of physicians and a practice-oriented approach in preparation for Generation Z
Source: Onkologe (Berl). 2022 May 2;55(6):447–54. [Article in German] doi: 10.1007/s00129-022-04942-5 (PMC9058740; doi:10.1007/s00129-022-04942-5)
Supplement: Supplementary file 1 [file 129_2022_4942_MOESM1_ESM.pdf]

F1 Welchem Geburtsjahrgang gehören Sie an?

Beantwortet: 122   Übersprungen: 0

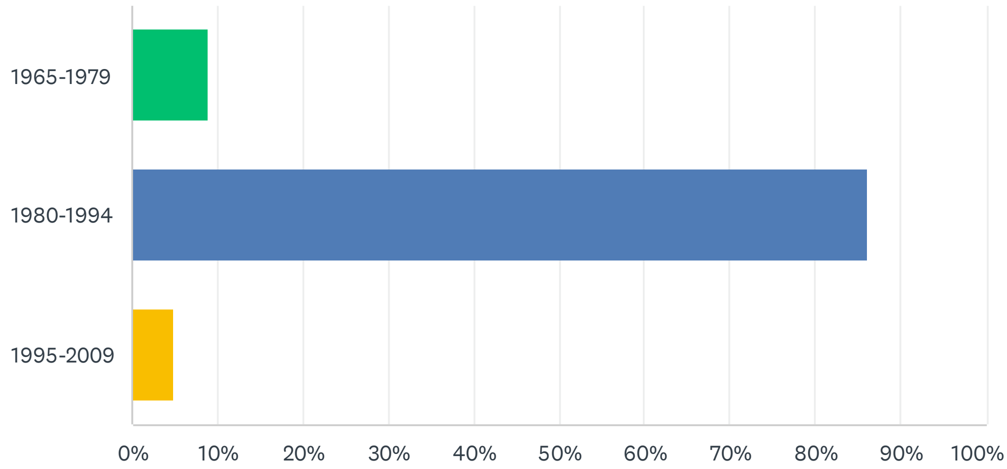

ANTWORTOPTIONEN	BEANTWORTUNGEN	
1965-1979	9.02%	11
1980-1994	86.07%	105
1995-2009	4.92%	6
GESAMT		122

F2 Welchem Geschlecht fühlen Sie sich am ehesten zugehörig?

Beantwortet: 122 Übersprungen: 0

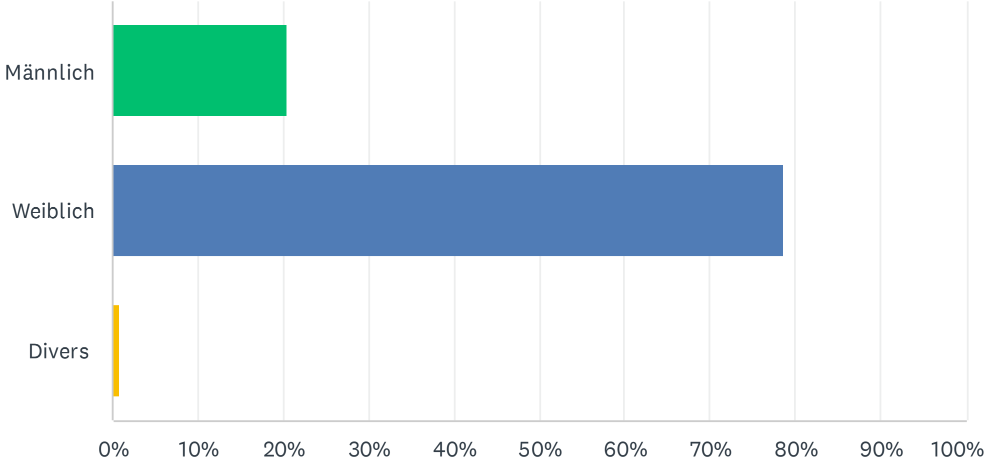

ANTWORTOPTIONEN	BEANTWORTUNGEN	
Männlich	20.49%	25
Weiblich	78.69%	96
Divers	0.82%	1
GESAMT		122

F3 In welchem Weiterbildungsjahr befinden Sie sich?

Beantwortet: 122   Übersprungen: 0

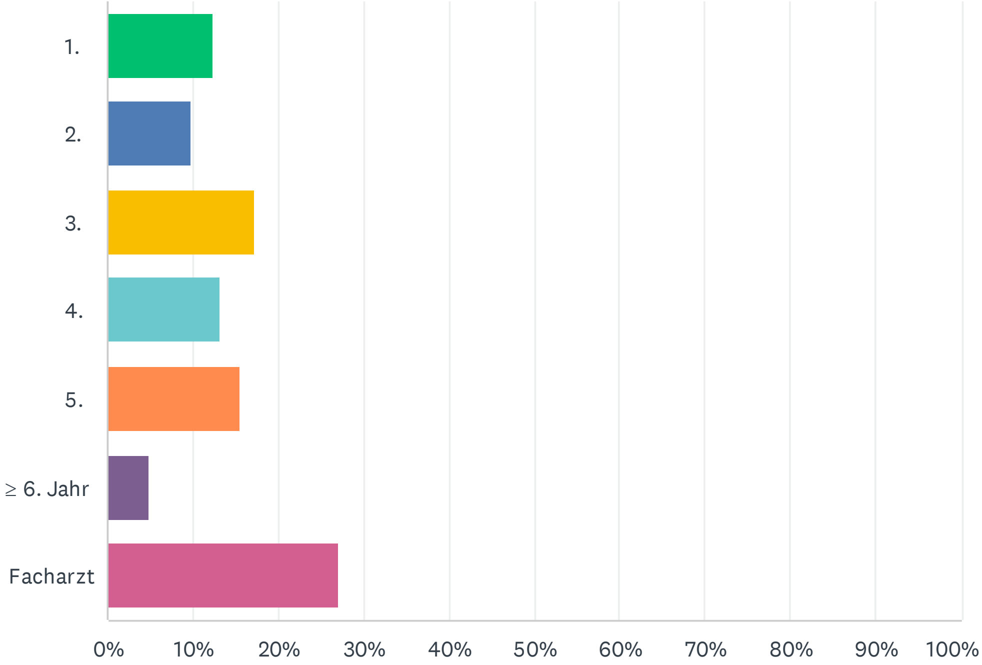

ANTWORTOPTIONEN	BEANTWORTUNGEN	
1.	12.30%	15
2.	9.84%	12
3.	17.21%	21
4.	13.11%	16
5.	15.57%	19
≥ 6. Jahr	4.92%	6
Facharzt	27.05%	33
GESAMT		122

F4 Haben Sie promoviert?

Beantwortet: 122   Übersprungen: 0

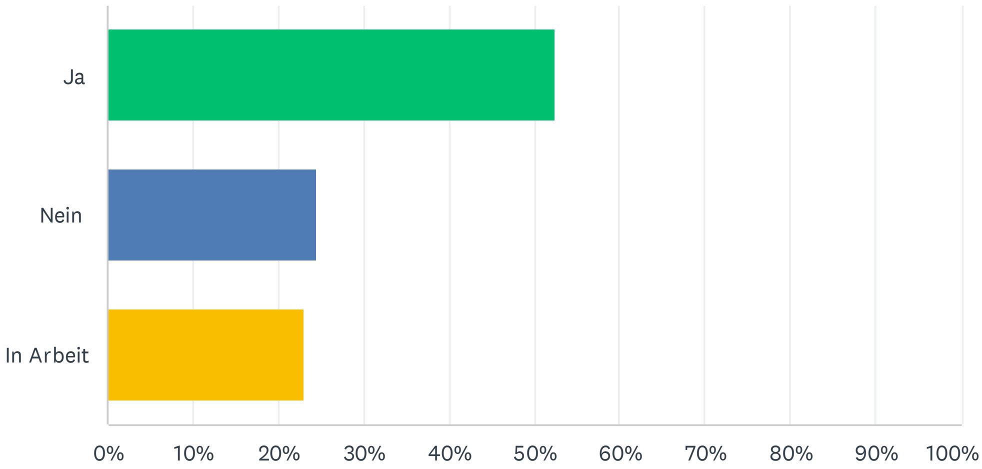

ANTWORTOPTIONEN	BEANTWORTUNGEN	
Ja	52.46%	64
Nein	24.59%	30
In Arbeit	22.95%	28
GESAMT		122

F5 Haben Sie bereits eine Familie gegründet bzw. haben Sie Kinder?

Beantwortet: 122   Übersprungen: 0

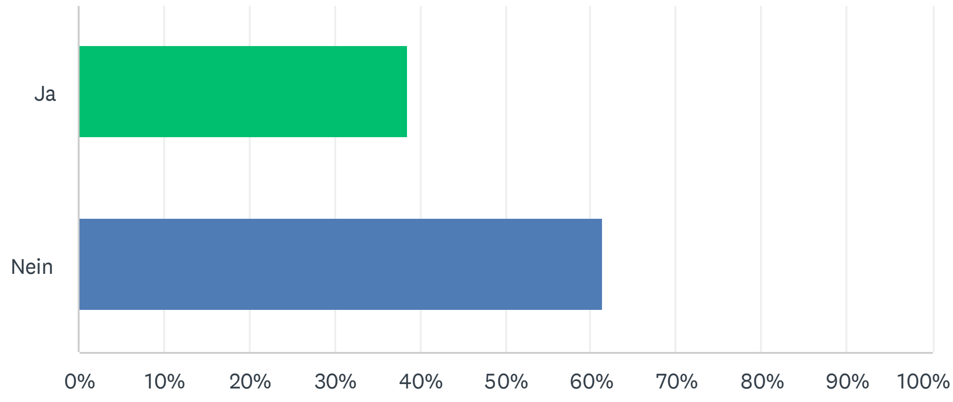

ANTWORTOPTIONEN	BEANTWORTUNGEN	
Ja	38.52%	47
Nein	61.48%	75
GESAMT		122

F6 Arbeiten Sie derzeit in Vollzeit oder Teilzeit?

Beantwortet: 122 Übersprungen: 0

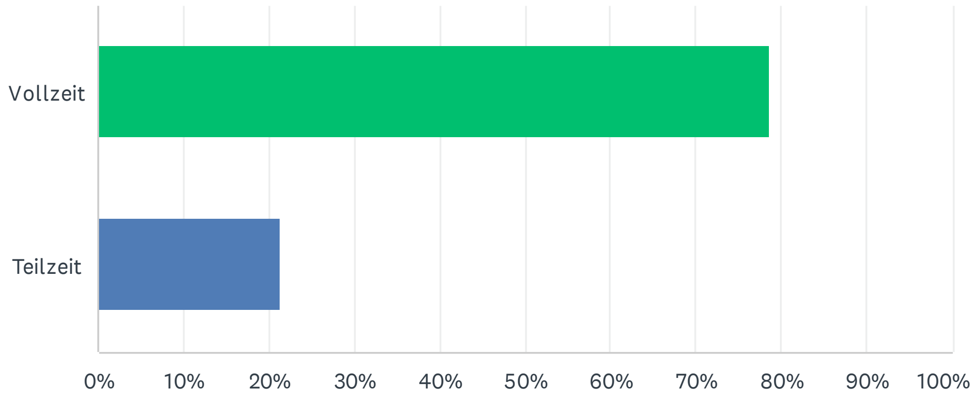

ANTWORTOPTIONEN	BEANTWORTUNGEN	
Vollzeit	78.69%	96
Teilzeit	21.31%	26
GESAMT		122

F7 Spezifizieren Sie bitte Ihre Arbeit in Teilzeit:

Beantwortet: 26 Übersprungen: 96

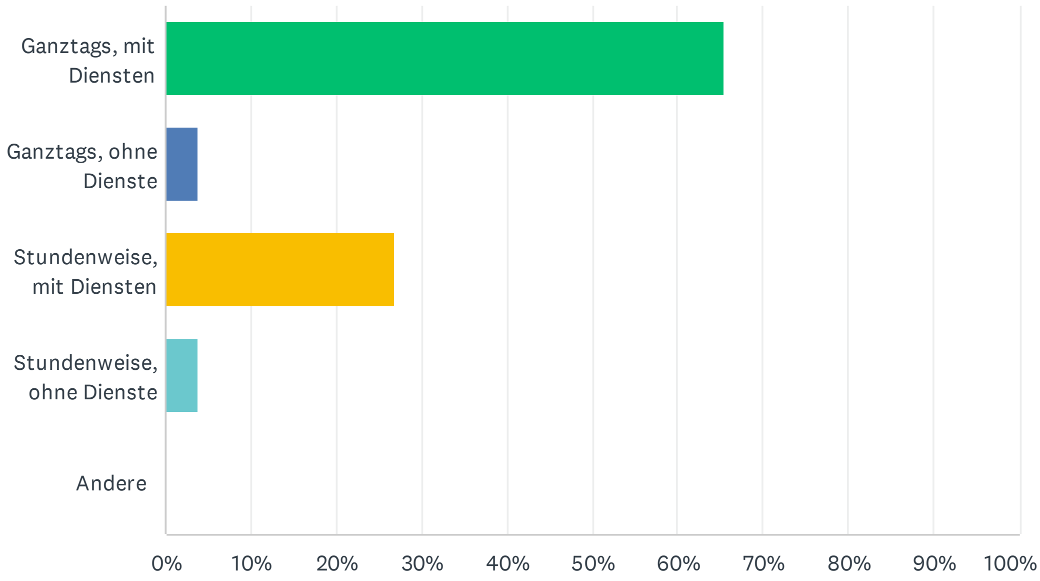

ANTWORTOPTIONEN	BEANTWORTUNGEN	
Ganztags, mit Diensten	65.38%	17
Ganztags, ohne Dienste	3.85%	1
Stundenweise, mit Diensten	26.92%	7
Stundenweise, ohne Dienste	3.85%	1
Andere	0.00%	0
GESAMT		26

F8 Arbeiten Sie an einer Universitätsklinik, einem akademischen Lehrkrankenhaus oder an einer kommunalen Klinik?

Beantwortet: 122 Übersprungen: 0

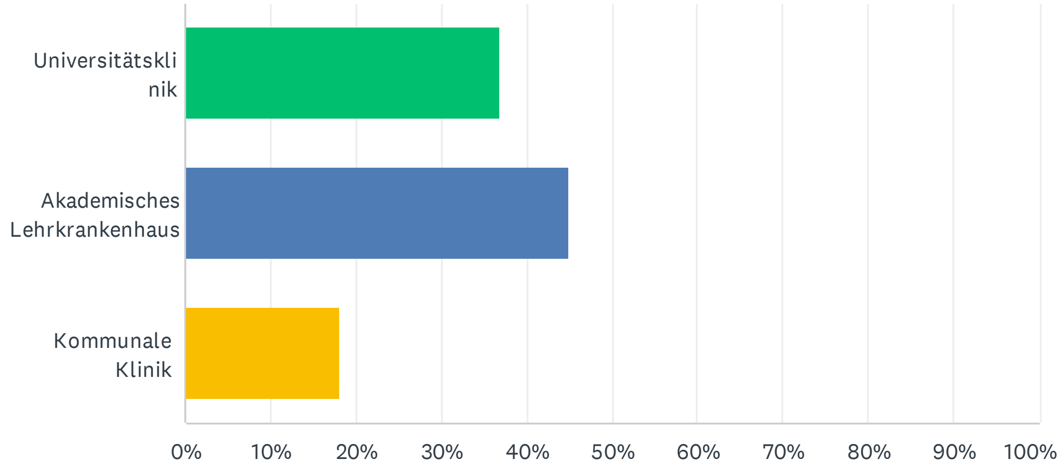

ANTWORTOPTIONEN	BEANTWORTUNGEN	
Universitätsklinik	36.89%	45
Akademisches Lehrkrankenhaus	45.08%	55
Kommunale Klinik	18.03%	22
GESAMT		122

F9 Wie beurteilen Sie Ihre Arbeitsbedingungen?

Beantwortet: 119 Übersprungen: 3

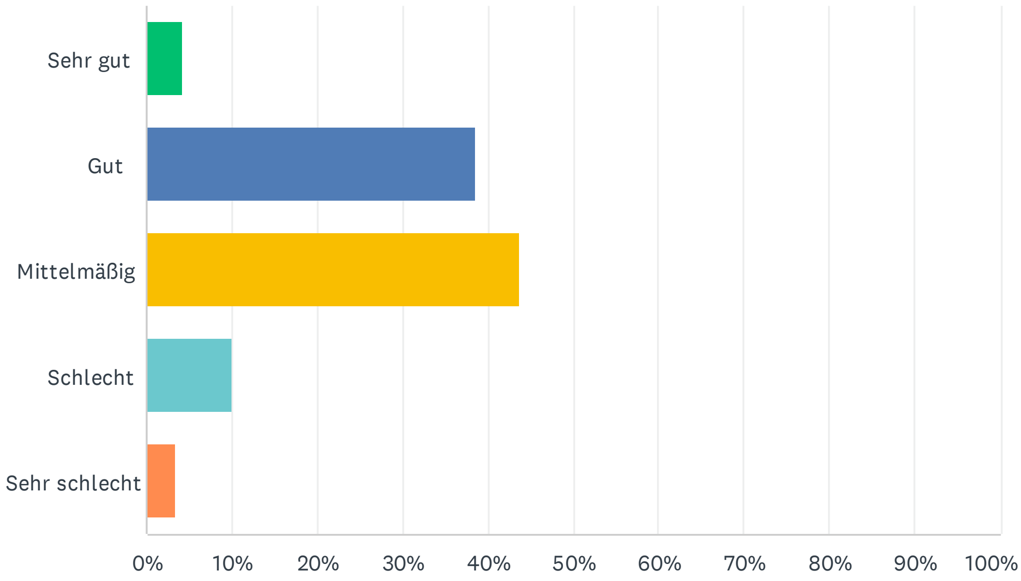

ANTWORTOPTIONEN	BEANTWORTUNGEN	
Sehr gut	4.20%	5
Gut	38.66%	46
Mittelmäßig	43.70%	52
Schlecht	10.08%	12
Sehr schlecht	3.36%	4
GESAMT		119

F10 Wie schätzen Sie Ihre subjektiv empfundene Arbeitsbelastung ein?

Beantwortet: 119   Übersprungen: 3

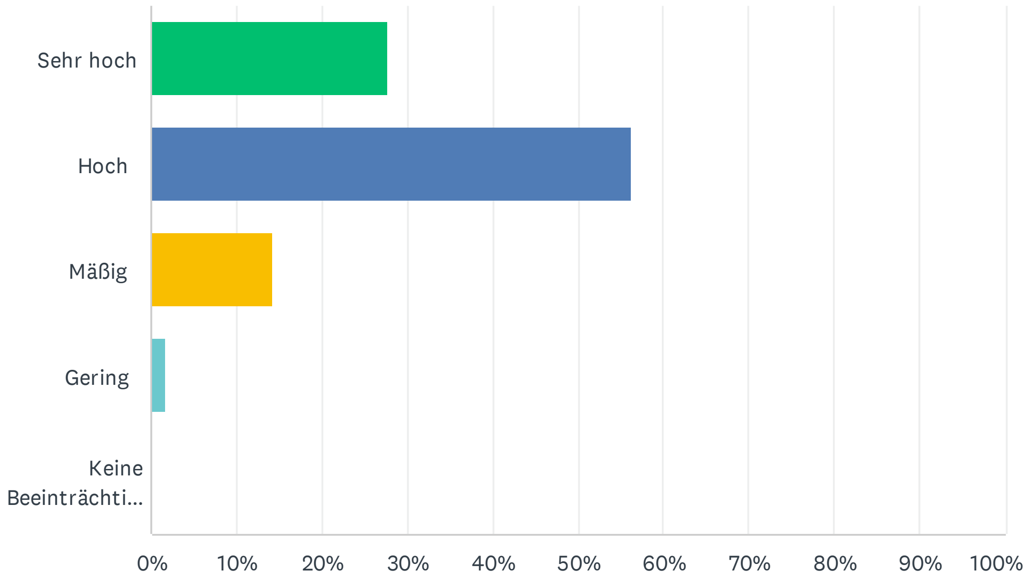

ANTWORTOPTIONEN	BEANTWORTUNGEN	
Sehr hoch	27.73%	33
Hoch	56.30%	67
Mäßig	14.29%	17
Gering	1.68%	2
Keine Beeinträchtigung	0.00%	0
GESAMT		119

F11 Wie beurteilen Sie Ihre Einarbeitung als Berufsanfänger bzw. zu Beginn einer neuen Rotation?

Beantwortet: 119   Übersprungen: 3

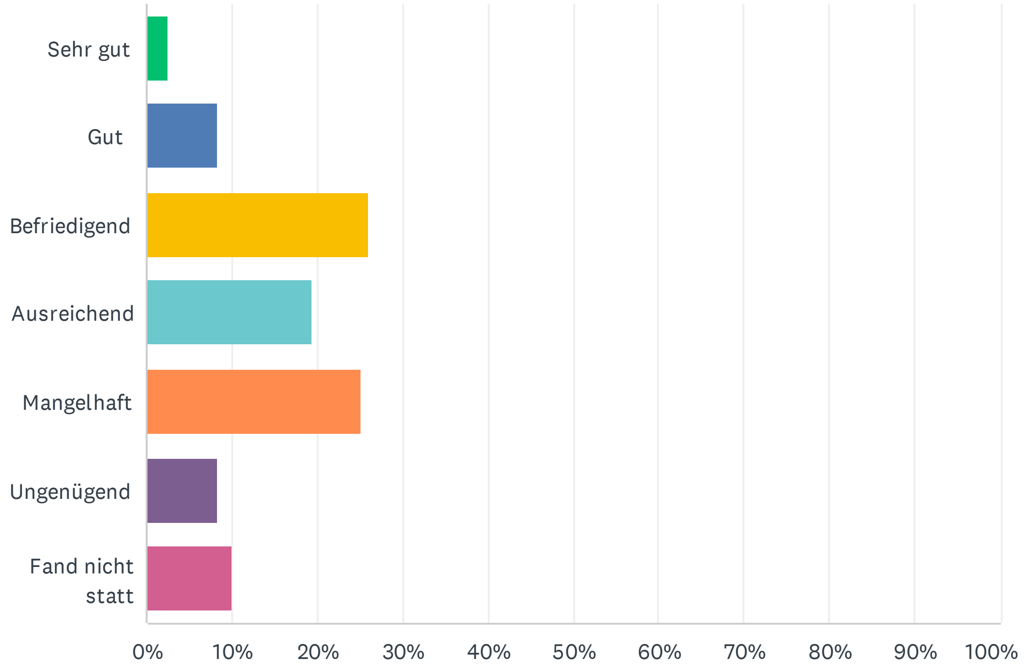

ANTWORTOPTIONEN	BEANTWORTUNGEN	
Sehr gut	2.52%	3
Gut	8.40%	10
Befriedigend	26.05%	31
Ausreichend	19.33%	23
Mangelhaft	25.21%	30
Ungenügend	8.40%	10
Fand nicht statt	10.08%	12
GESAMT		119

F12 Sind Sie zufrieden mit Ihrem derzeitigen Einkommen?

Beantwortet: 119 Übersprungen: 3

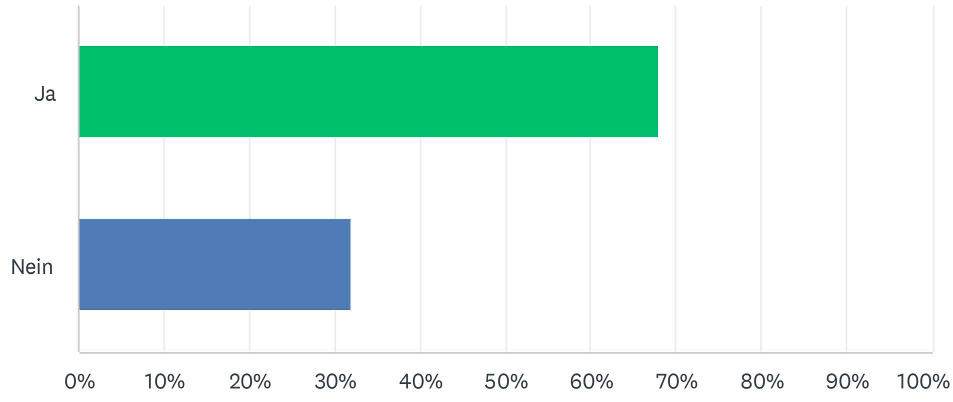

ANTWORTOPTIONEN	BEANTWORTUNGEN	
Ja	68.07%	81
Nein	31.93%	38
GESAMT		119

F13 Halten Sie Ihr Einkommen in Bezug auf Ihre geleistete Arbeit und Verantwortung für angemessen?

Beantwortet: 120   Übersprungen: 2

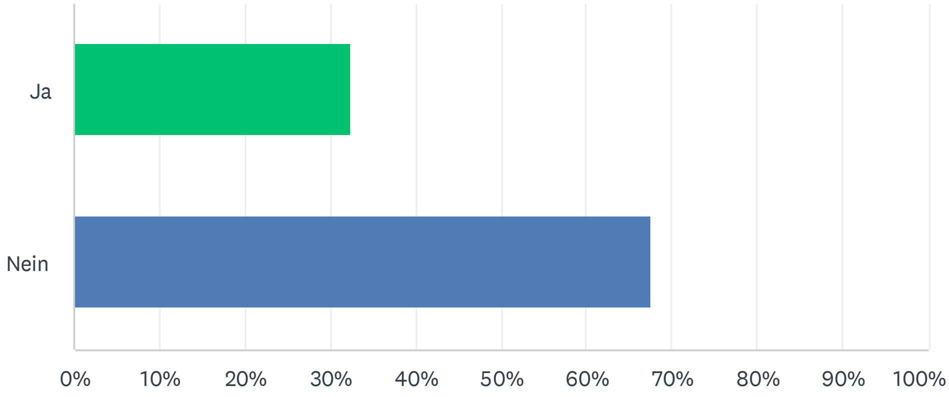

ANTWORTOPTIONEN	BEANTWORTUNGEN	
Ja	32.50%	39
Nein	67.50%	81
GESAMT		120

F14 Wie hoch fällt die tariflich vereinbarte Wochenarbeitszeit Ihrer Vollzeitstelle aus?

Beantwortet: 120 Übersprungen: 2

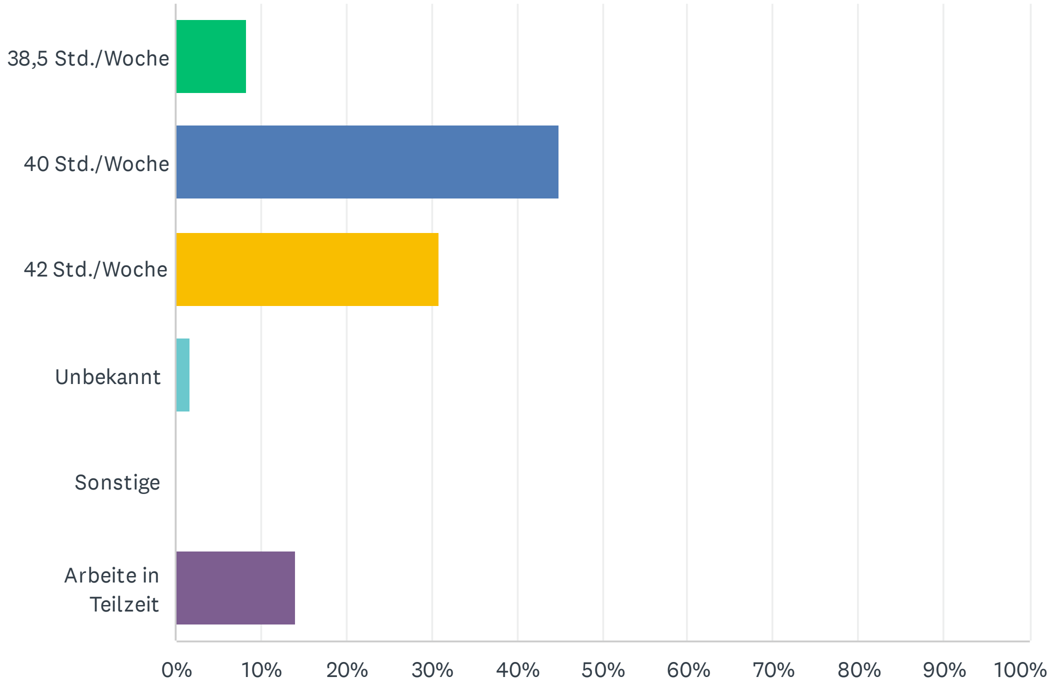

ANTWORTOPTIONEN	BEANTWORTUNGEN	
38,5 Std./Woche	8.33%	10
40 Std./Woche	45.00%	54
42 Std./Woche	30.83%	37
Unbekannt	1.67%	2
Sonstige	0.00%	0
Arbeite in Teilzeit	14.17%	17
GESAMT		120

F15 Wie hoch fallen Ihre durchschnittlichen wöchentlichen Arbeitsstunden aus (exkl. Dienste, inkl. Überstunden)?

Beantwortet: 120   Übersprungen: 2

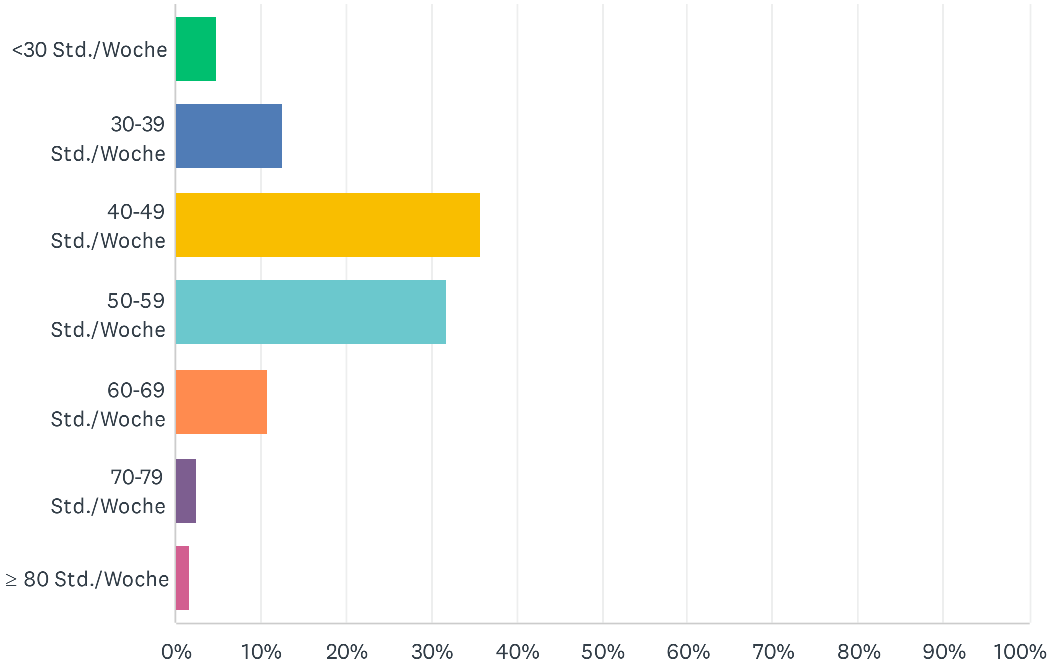

ANTWORTOPTIONEN	BEANTWORTUNGEN	
<30 Std./Woche	5.00%	6
30-39 Std./Woche	12.50%	15
40-49 Std./Woche	35.83%	43
50-59 Std./Woche	31.67%	38
60-69 Std./Woche	10.83%	13
70-79 Std./Woche	2.50%	3
≥ 80 Std./Woche	1.67%	2
GESAMT		120

F16 Welche durchschnittliche Wochenarbeitszeit bevorzugen Sie?

Beantwortet: 119 Übersprungen: 3

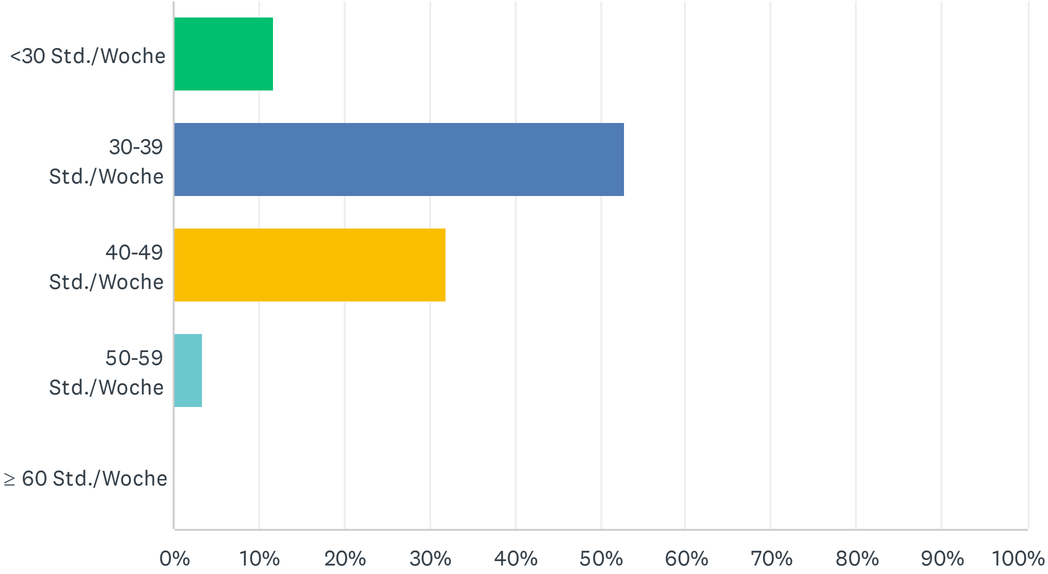

ANTWORTOPTIONEN	BEANTWORTUNGEN	
<30 Std./Woche	11.76%	14
30-39 Std./Woche	52.94%	63
40-49 Std./Woche	31.93%	38
50-59 Std./Woche	3.36%	4
≥ 60 Std./Woche	0.00%	0
GESAMT		119

F17 Präferieren Sie flexible oder feste Arbeitszeiten?

Beantwortet: 119 Übersprungen: 3

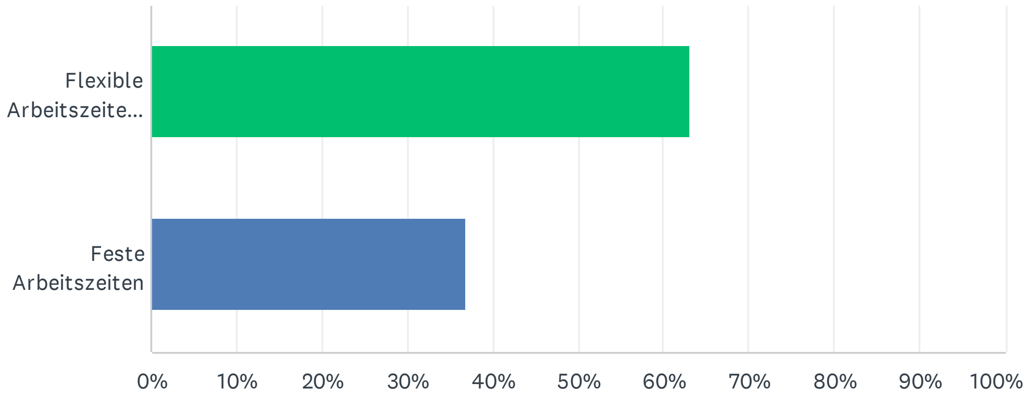

ANTWORTOPTIONEN	BEANTWORTUNGEN	
Flexible Arbeitszeiten (z.B. Gleitzeit)	63.03%	75
Feste Arbeitszeiten	36.97%	44
GESAMT		119

F18 Halten Sie Ihre vorgeschriebenen Arbeitspausen ein?

Beantwortet: 120 Übersprungen: 2

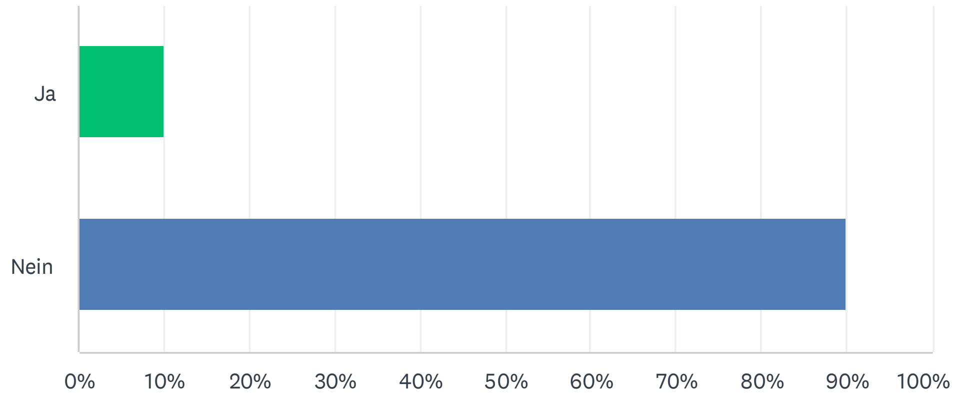

ANTWORTOPTIONEN	BEANTWORTUNGEN	
Ja	10.00%	12
Nein	90.00%	108
GESAMT		120

F19 Verlassen Sie für die Arbeitspausen Ihren Arbeitsplatz?

Beantwortet: 12 Übersprungen: 110

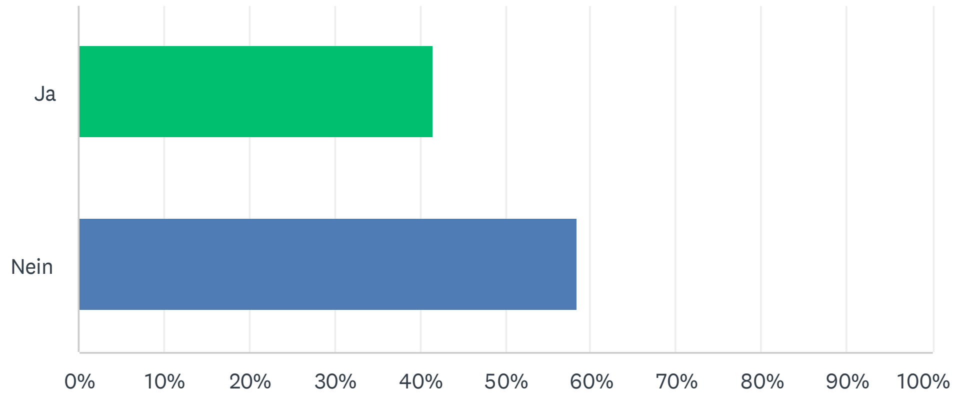

ANTWORTOPTIONEN	BEANTWORTUNGEN	
Ja	41.67%	5
Nein	58.33%	7
GESAMT		12

F20 Leisten Sie regelmäßig Überstunden?

Beantwortet: 119 Übersprungen: 3

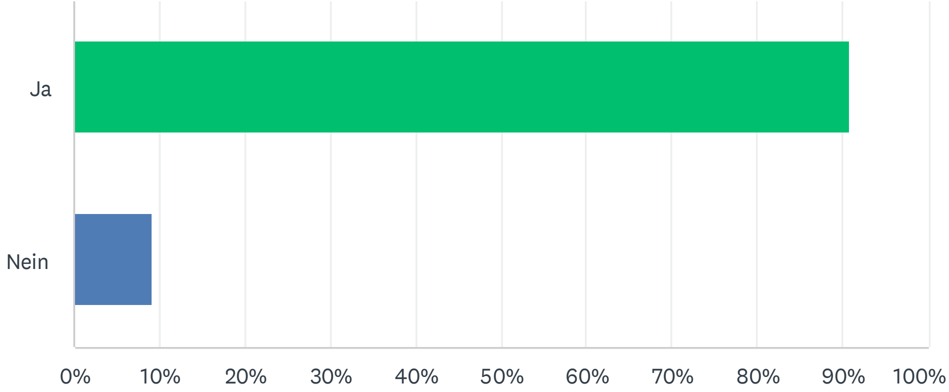

ANTWORTOPTIONEN	BEANTWORTUNGEN	
Ja	90.76%	108
Nein	9.24%	11
GESAMT		119

F21 Wie hoch fallen Ihre durchschnittlichen wöchentlichen Überstunden aus?

Beantwortet: 119 Übersprungen: 3

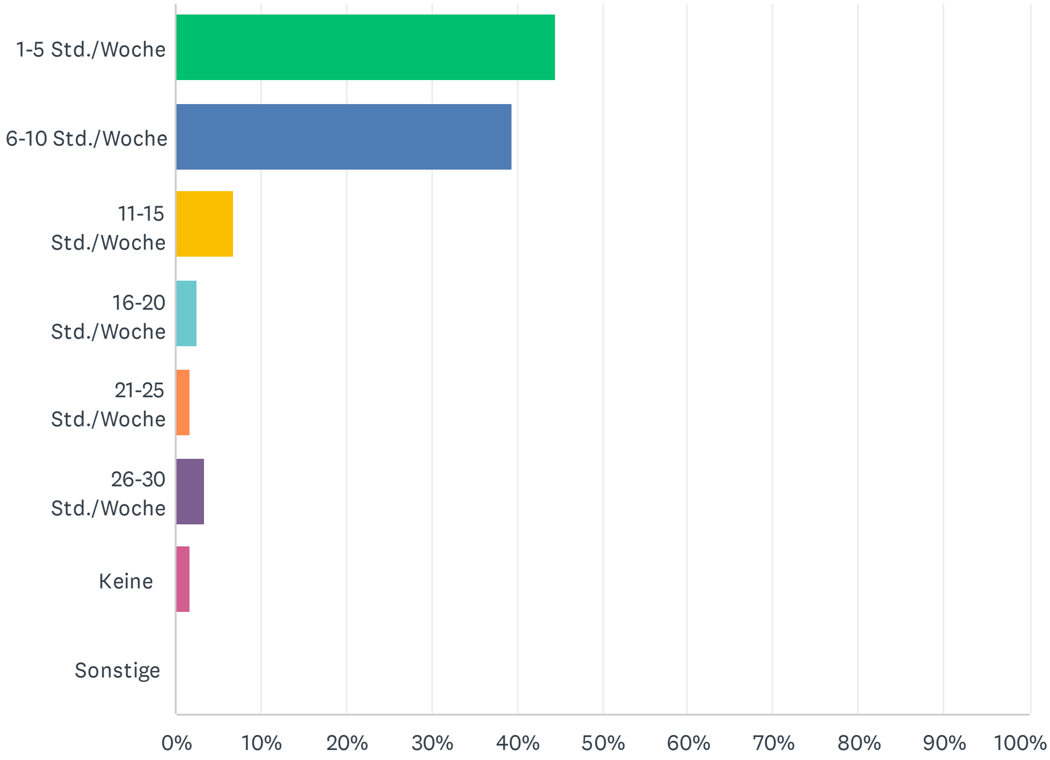

ANTWORTOPTIONEN	BEANTWORTUNGEN	
1-5 Std./Woche	44.54%	53
6-10 Std./Woche	39.50%	47
11-15 Std./Woche	6.72%	8
16-20 Std./Woche	2.52%	3
21-25 Std./Woche	1.68%	2
26-30 Std./Woche	3.36%	4
Keine	1.68%	2
Sonstige	0.00%	0
GESAMT		119

F22 Was sind die Gründe der anfallenden Überstunden (Mehrfachnennung)?

Beantwortet: 119 Übersprungen: 3

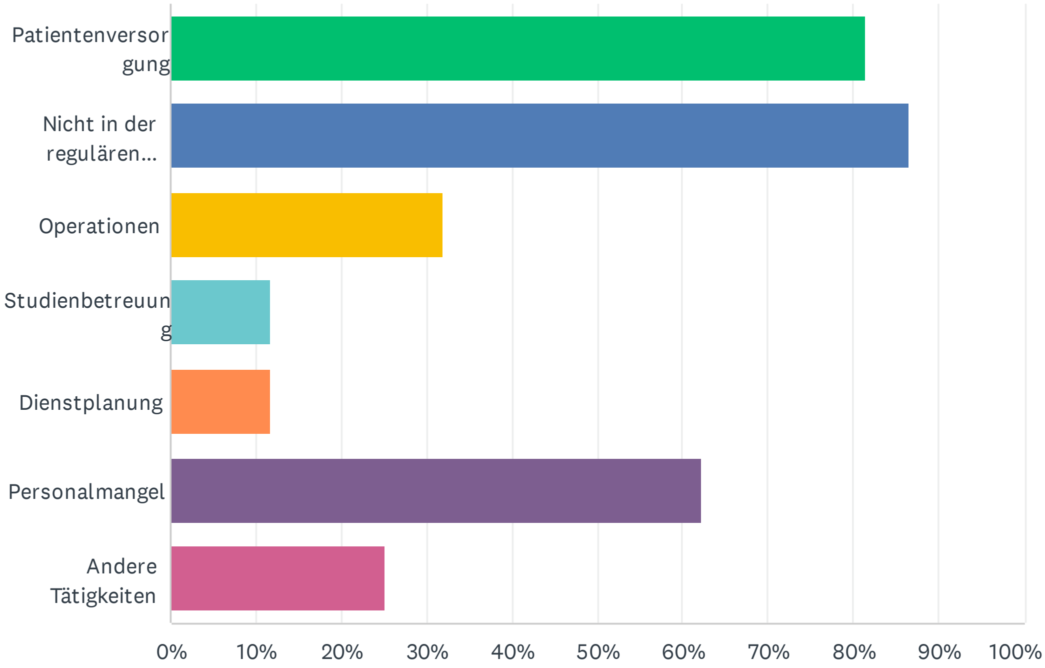

ANTWORTOPTIONEN	BEANTWORTUNGEN	
Patientenversorgung	81.51%	97
Nicht in der regulären Arbeitszeit erledigte Dokumentation	86.55%	103
Operationen	31.93%	38
Studienbetreuung	11.76%	14
Dienstplanung	11.76%	14
Personalmangel	62.18%	74
Andere Tätigkeiten	25.21%	30
Befragte insgesamt: 119		

F23 Werden die geleisteten Überstunden explizit angeordnet?

Beantwortet: 119 Übersprungen: 3

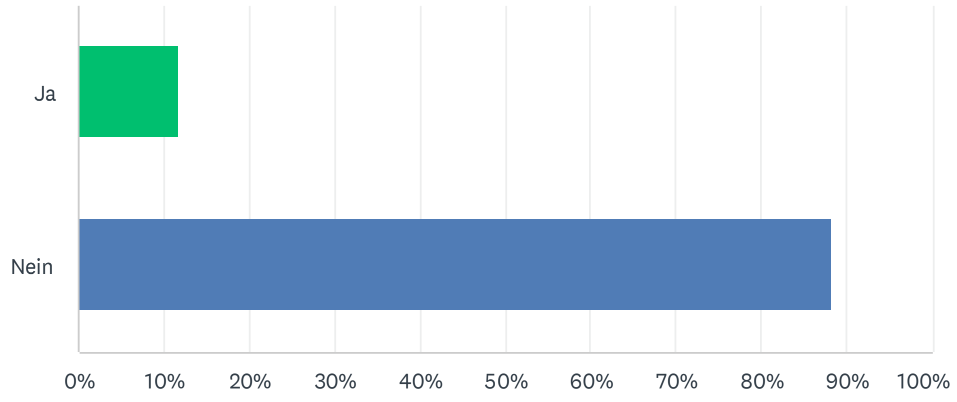

ANTWORTOPTIONEN	BEANTWORTUNGEN	
Ja	11.76%	14
Nein	88.24%	105
GESAMT		119

F24 Besteht die Möglichkeit, sich Überstunden auszahlen zu lassen?

Beantwortet: 119 Übersprungen: 3

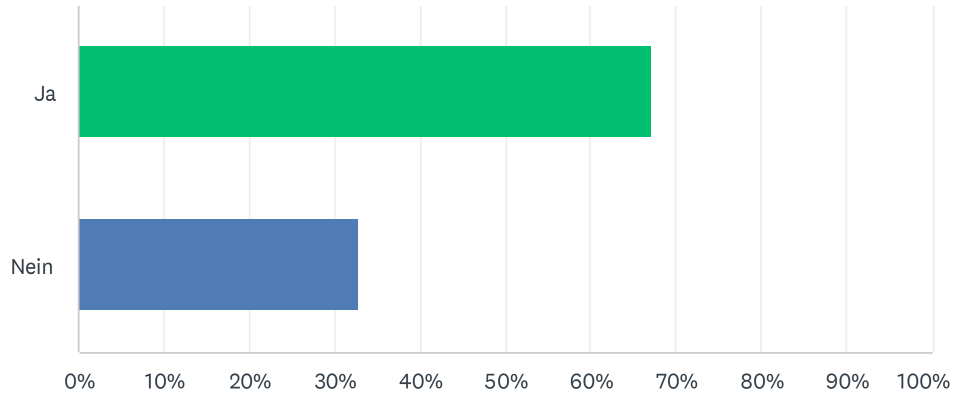

ANTWORTOPTIONEN	BEANTWORTUNGEN	
Ja	67.23%	80
Nein	32.77%	39
GESAMT		119

F25 Besteht die Möglichkeit, für geleistete Überstunden Freizeitausgleich zu erhalten?

Beantwortet: 118 Übersprungen: 4

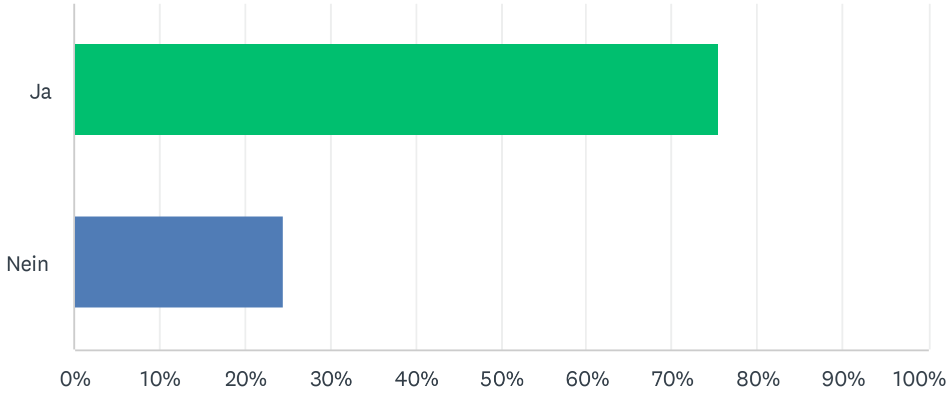

ANTWORTOPTIONEN	BEANTWORTUNGEN	
Ja	75.42%	89
Nein	24.58%	29
GESAMT		118

F26 Besteht die Möglichkeit, nicht eingehaltene Pausen als Überstunden zu dokumentieren?

Beantwortet: 119 Übersprungen: 3

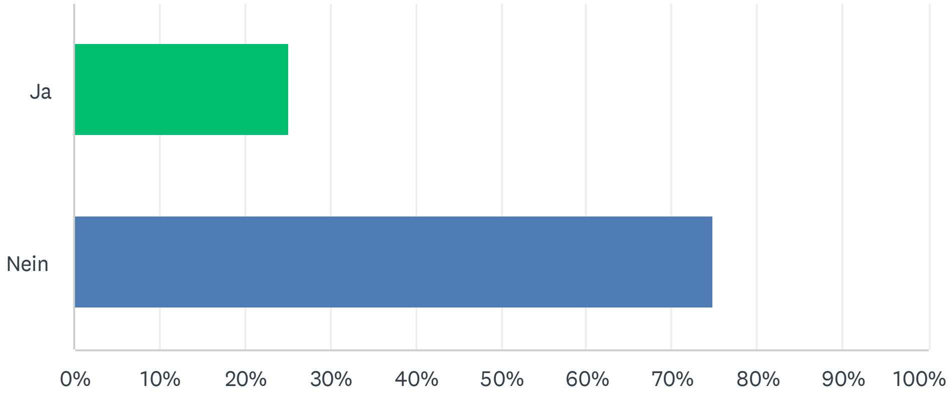

ANTWORTOPTIONEN	BEANTWORTUNGEN	
Ja	25.21%	30
Nein	74.79%	89
GESAMT		119

F27 Wie hoch schätzen Sie den Anteil delegierbarer, nicht-ärztlicher Tätigkeiten ein?

Beantwortet: 119 Übersprungen: 3

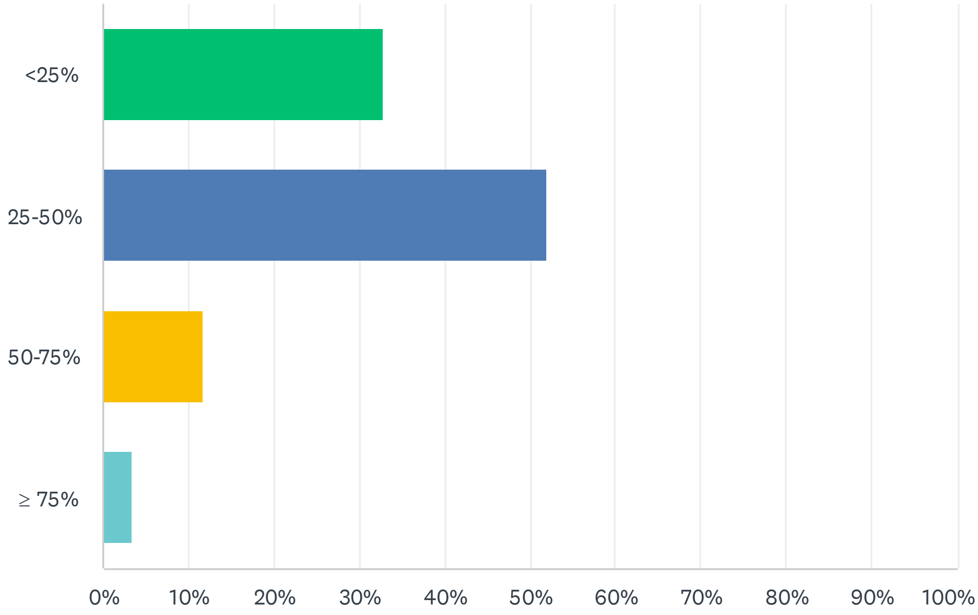

ANTWORTOPTIONEN	BEANTWORTUNGEN	
<25%	32.77%	39
25-50%	52.10%	62
50-75%	11.76%	14
≥ 75%	3.36%	4
GESAMT		119

F28 Wie groß schätzen Sie Ihren Anteil der Dokumentation in Bezug auf Ihre tägliche Arbeitszeit ein?

Beantwortet: 119   Übersprungen: 3

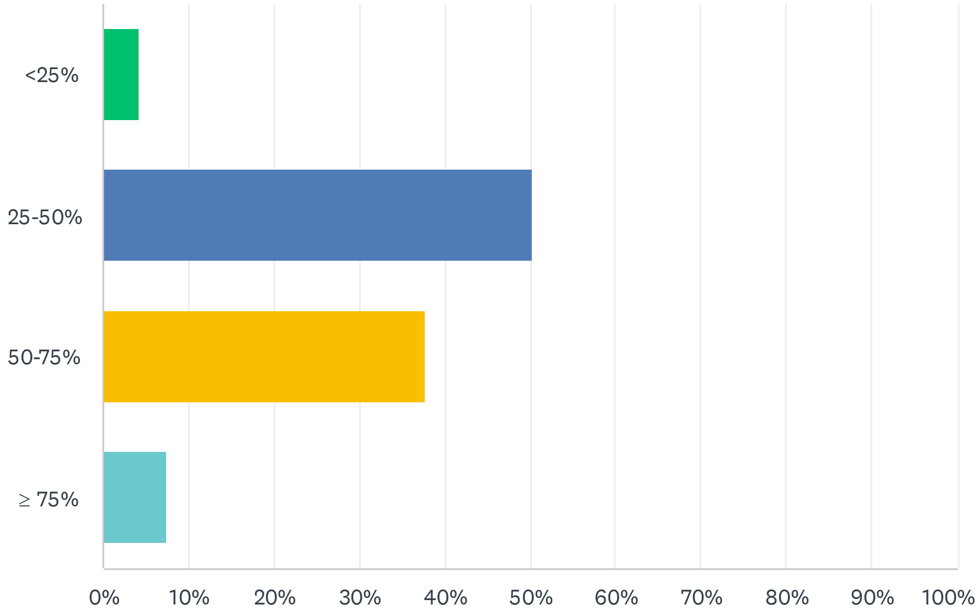

ANTWORTOPTIONEN	BEANTWORTUNGEN	
<25%	4.20%	5
25-50%	50.42%	60
50-75%	37.82%	45
≥ 75%	7.56%	9
GESAMT		119

F29 Wie zufrieden sind Sie mit dem Führungsstil Ihres Chefarztes?

Beantwortet: 119   Übersprungen: 3

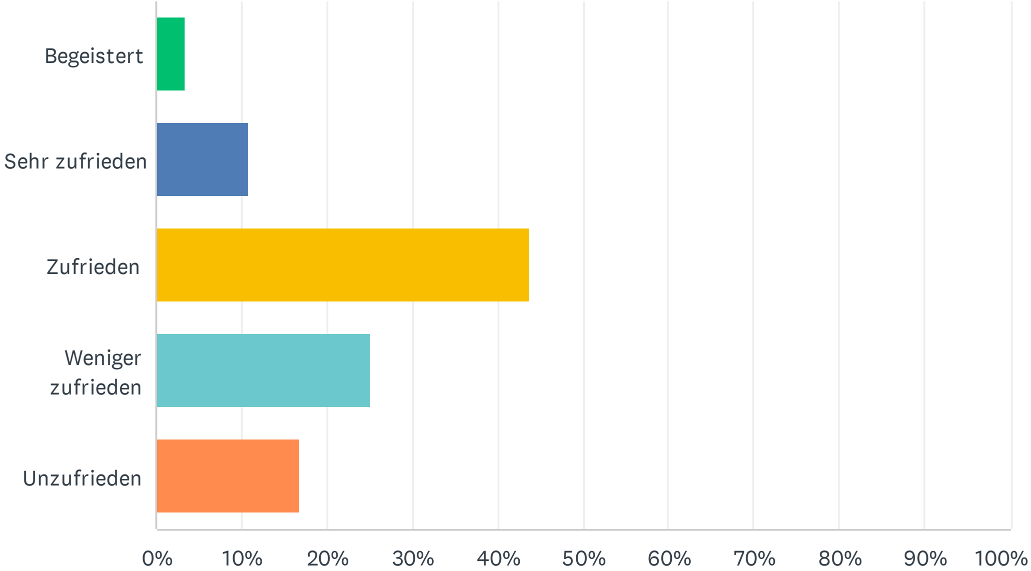

ANTWORTOPTIONEN	BEANTWORTUNGEN	
Begeistert	3.36%	4
Sehr zufrieden	10.92%	13
Zufrieden	43.70%	52
Weniger zufrieden	25.21%	30
Unzufrieden	16.81%	20
GESAMT		119

F30 Stellt Ihr Chefarzt für Sie eine Vorbildfunktion dar?

Beantwortet: 119 Übersprungen: 3

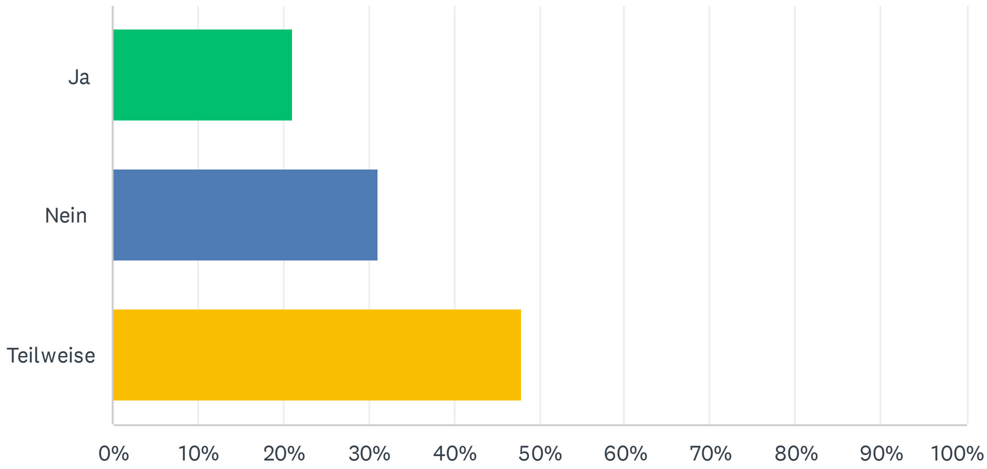

ANTWORTOPTIONEN	BEANTWORTUNGEN	
Ja	21.01%	25
Nein	31.09%	37
Teilweise	47.90%	57
GESAMT		119

F31 Stellen Ihre Oberärzte für Sie eine Vorbildfunktion dar?

Beantwortet: 119 Übersprungen: 3

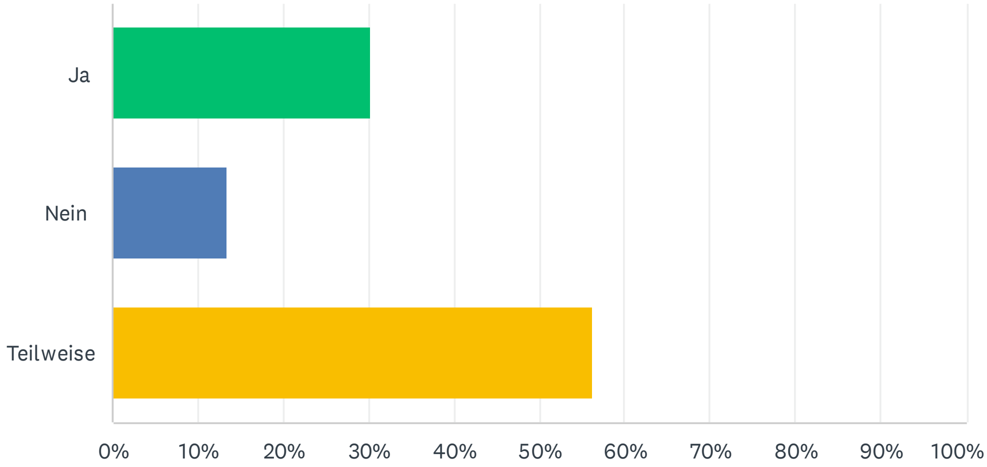

ANTWORTOPTIONEN	BEANTWORTUNGEN	
Ja	30.25%	36
Nein	13.45%	16
Teilweise	56.30%	67
GESAMT		119

F32 Finden regelmäßige Chefarztvisiten statt?

Beantwortet: 119   Übersprungen: 3

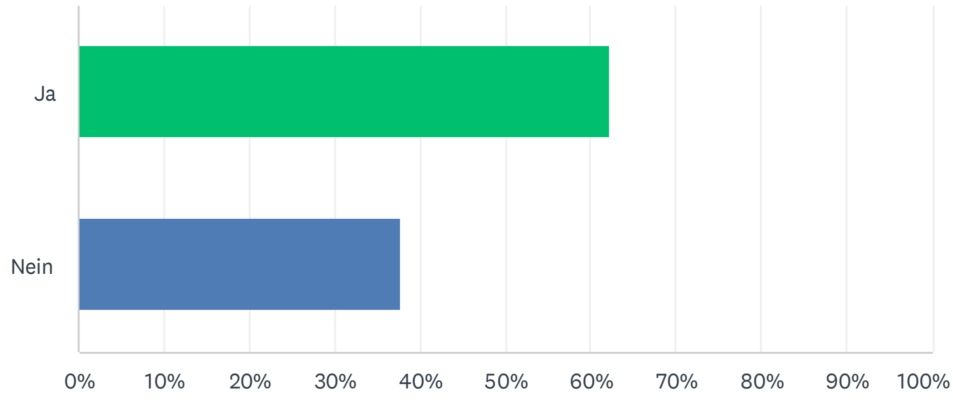

ANTWORTOPTIONEN	BEANTWORTUNGEN	
Ja	62.18%	74
Nein	37.82%	45
GESAMT		119

F33 Finden regelmäßige Oberarztvisiten am Patientenbett statt?

Beantwortet: 119   Übersprungen: 3

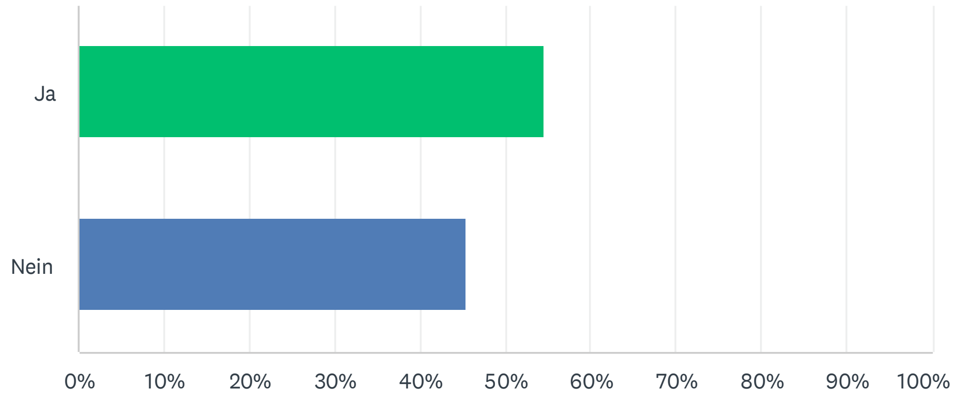

ANTWORTOPTIONEN	BEANTWORTUNGEN	
Ja	54.62%	65
Nein	45.38%	54
GESAMT		119

F34 Wünschen Sie sich regelmäßige Oberarzt- bzw. Chefarztvisiten?

Beantwortet: 119 Übersprungen: 3

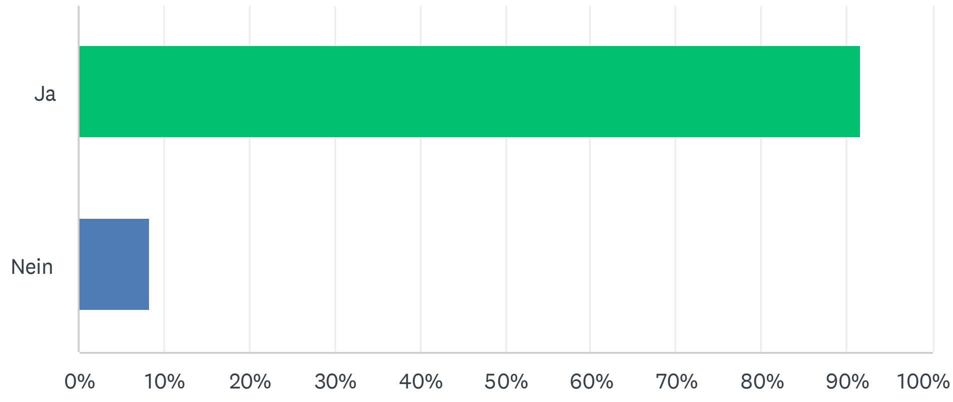

ANTWORTOPTIONEN	BEANTWORTUNGEN	
Ja	91.60%	109
Nein	8.40%	10
GESAMT		119

F35 Wie schätzen Sie den Stellenwert einer Promotion ein?

Beantwortet: 119 Übersprungen: 3

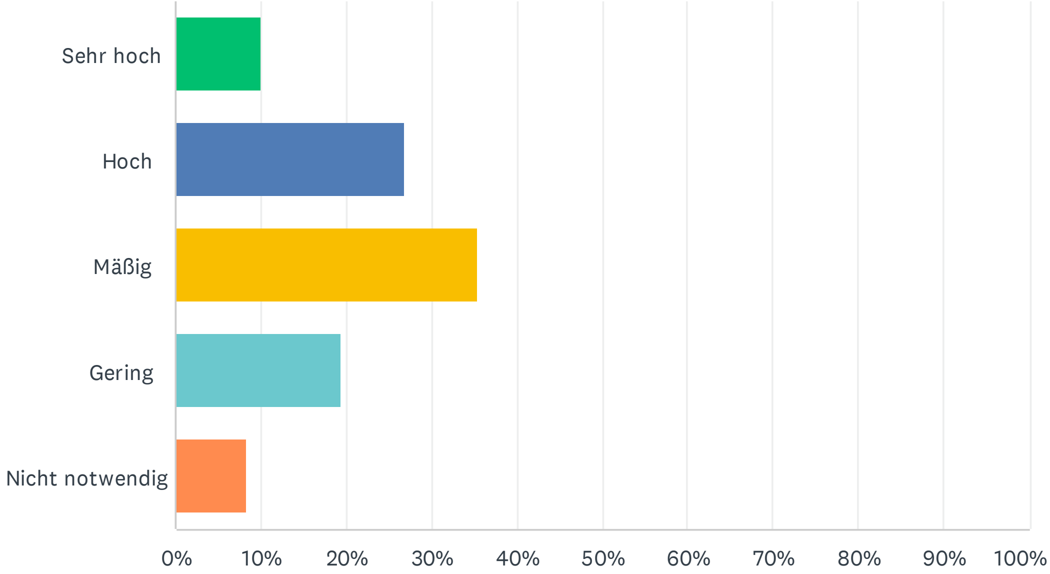

ANTWORTOPTIONEN	BEANTWORTUNGEN	
Sehr hoch	10.08%	12
Hoch	26.89%	32
Mäßig	35.29%	42
Gering	19.33%	23
Nicht notwendig	8.40%	10
GESAMT		119

F36 Wie schätzen Sie den Stellenwert des wissenschaftlichen Arbeitens ein?

Beantwortet: 116 Übersprungen: 6

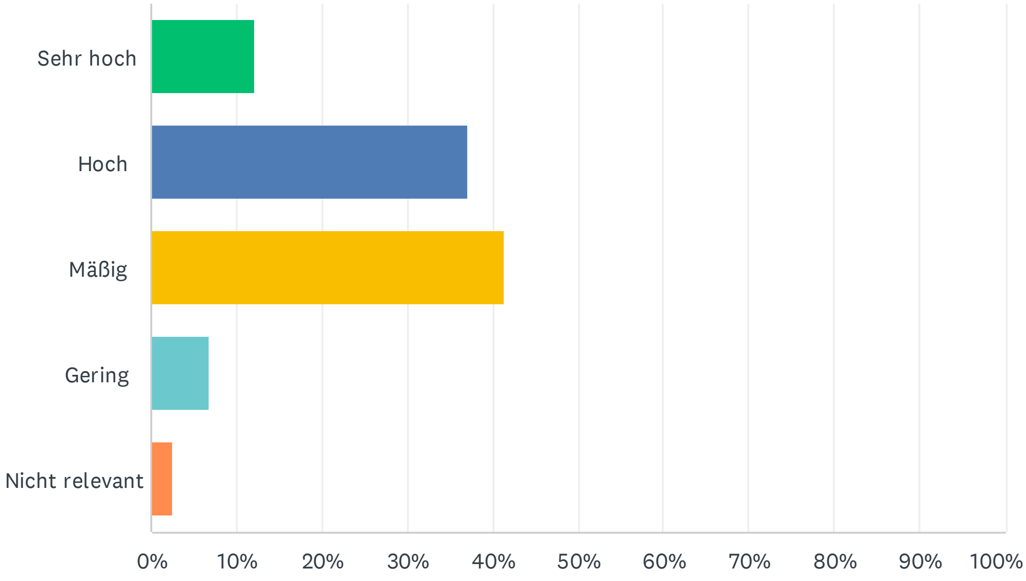

ANTWORTOPTIONEN	BEANTWORTUNGEN	
Sehr hoch	12.07%	14
Hoch	37.07%	43
Mäßig	41.38%	48
Gering	6.90%	8
Nicht relevant	2.59%	3
GESAMT		116

F37 Erhalten Sie an Ihrer Klinik ausreichend Freiraum, wissenschaftlich tätig zu werden?

Beantwortet: 119   Übersprungen: 3

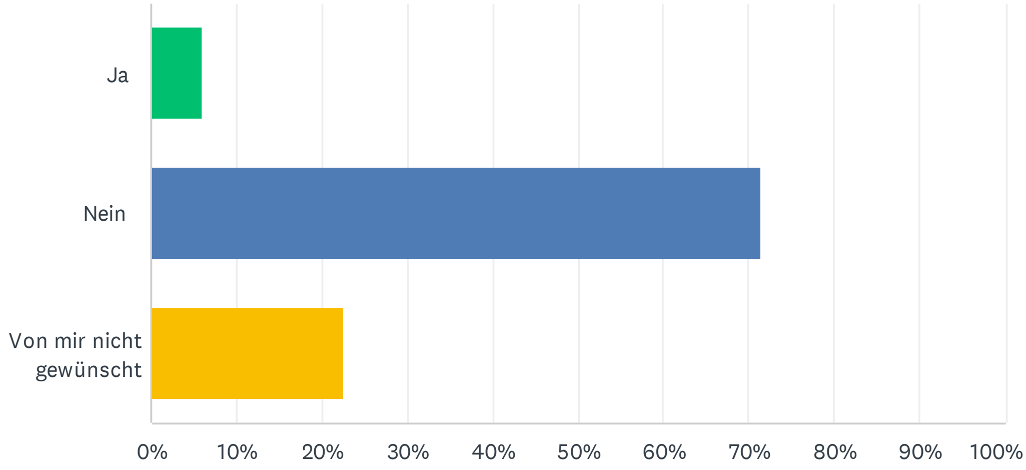

ANTWORTOPTIONEN	BEANTWORTUNGEN	
Ja	5.88%	7
Nein	71.43%	85
Von mir nicht gewünscht	22.69%	27
GESAMT		119

F38 Fühlen Sie sich als Arzt in Weiterbildung mit der durch Sie erbrachten Arbeit in Ihrer Klinik wertgeschätzt?

Beantwortet: 118 Übersprungen: 4

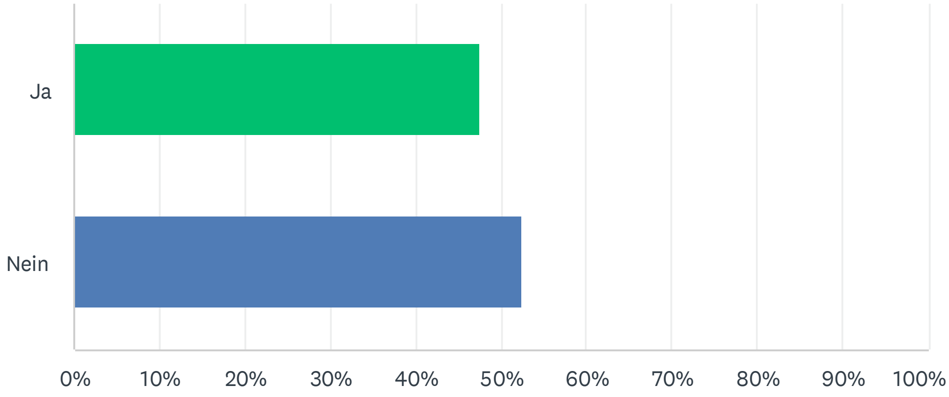

ANTWORTOPTIONEN	BEANTWORTUNGEN	
Ja	47.46%	56
Nein	52.54%	62
GESAMT		118

F39 Streben Sie im Verlauf eine Klinikanstellung oder eine spätere Niederlassung an?

Beantwortet: 119 Übersprungen: 3

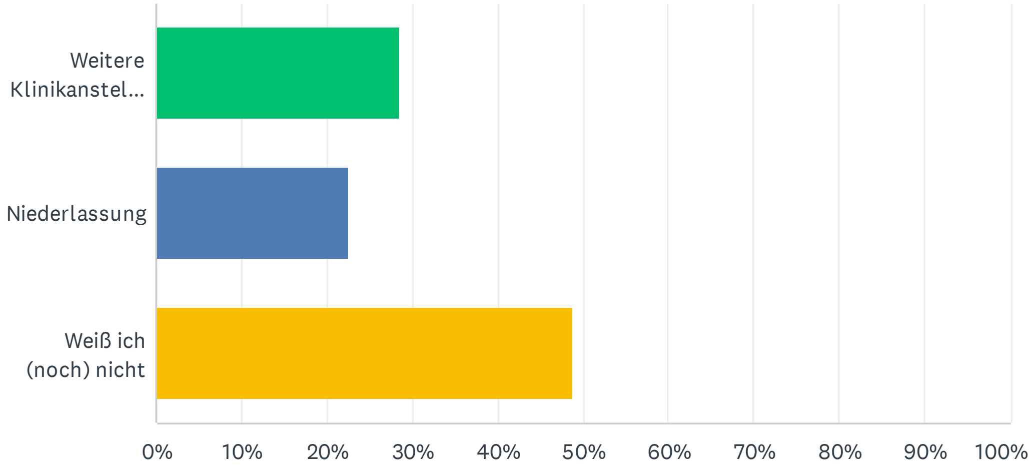

ANTWORTOPTIONEN	BEANTWORTUNGEN	
Weitere Klinikanstellung	28.57%	34
Niederlassung	22.69%	27
Weiß ich (noch) nicht	48.74%	58
GESAMT		119

F40 Sind Sie zufrieden mit Ihrer aktuellen beruflichen Situation?

Beantwortet: 119 Übersprungen: 3

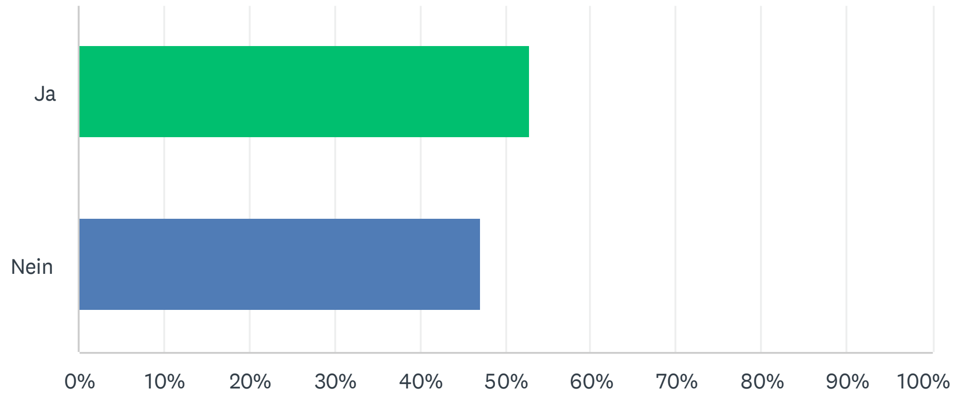

ANTWORTOPTIONEN	BEANTWORTUNGEN	
Ja	52.94%	63
Nein	47.06%	56
GESAMT		119

F41 Nennen Sie, bei Unzufriedenheit, einen bzw. mehrere Gründe, die zu dieser Unzufriedenheit beitragen:

Beantwortet: 56 Übersprungen: 66

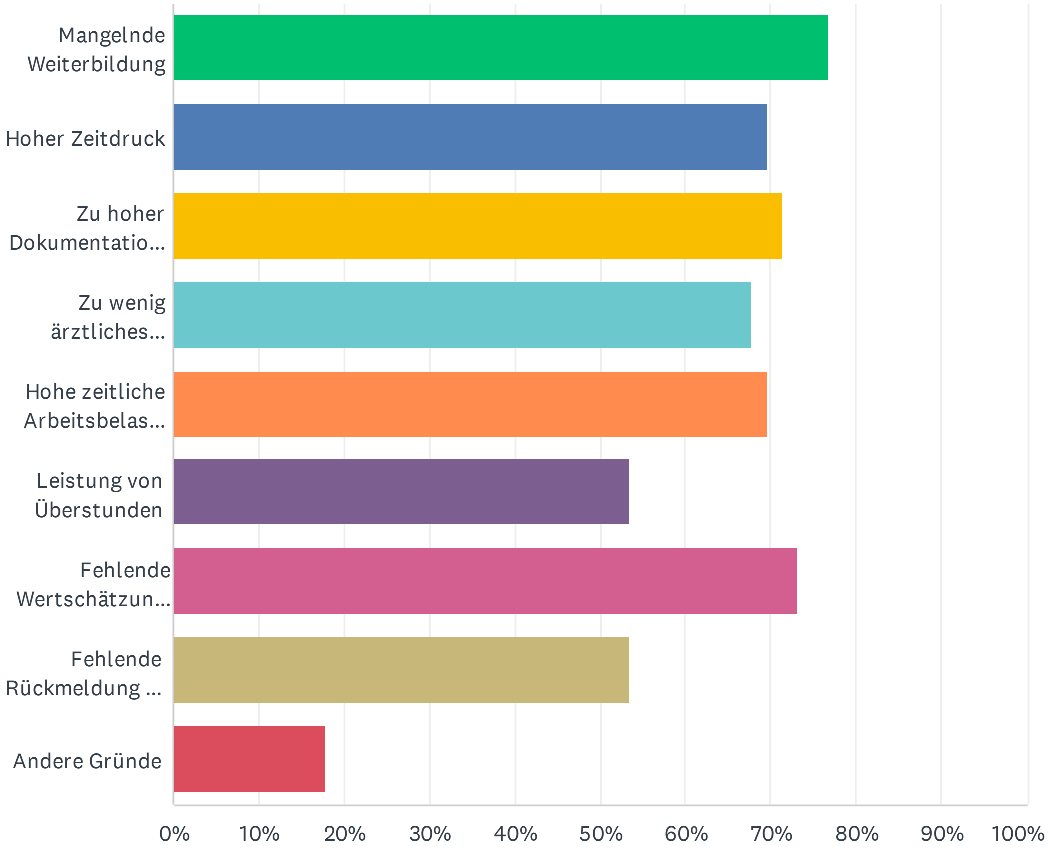

ANTWORTOPTIONEN	BEANTWORTUNGEN	
Mangelnde Weiterbildung	76.79%	43
Hoher Zeitdruck	69.64%	39
Zu hoher Dokumentationsaufwand	71.43%	40
Zu wenig ärztliches Personal in Relation zur Arbeit	67.86%	38
Hohe zeitliche Arbeitsbelastung	69.64%	39
Leistung von Überstunden	53.57%	30
Fehlende Wertschätzung der eigenen Arbeit	73.21%	41
Fehlende Rückmeldung der weiterbildenden Ärzte zur erbrachten Arbeitsleistung	53.57%	30
Andere Gründe	17.86%	10
Befragte insgesamt: 56		

F42 Wie würden Sie die kollegiale Zusammenarbeit bewerten?

Beantwortet: 117 Übersprungen: 5

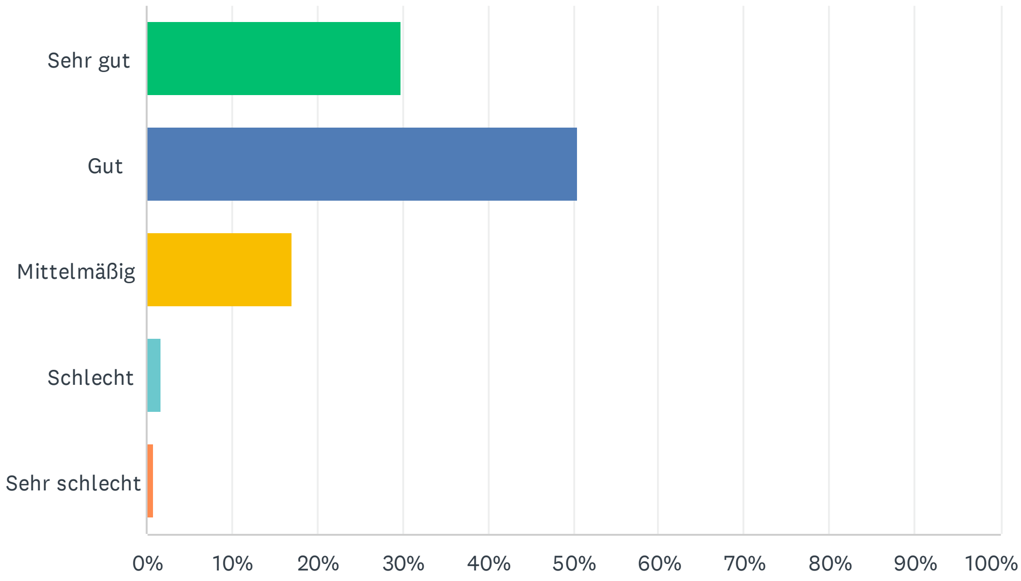

ANTWORTOPTIONEN	BEANTWORTUNGEN	
Sehr gut	29.91%	35
Gut	50.43%	59
Mittelmäßig	17.09%	20
Schlecht	1.71%	2
Sehr schlecht	0.85%	1
GESAMT		117

F43 Leidet Ihre kollegiale Zusammenarbeit unter der Arbeitsbelastung?

Beantwortet: 118 Übersprungen: 4

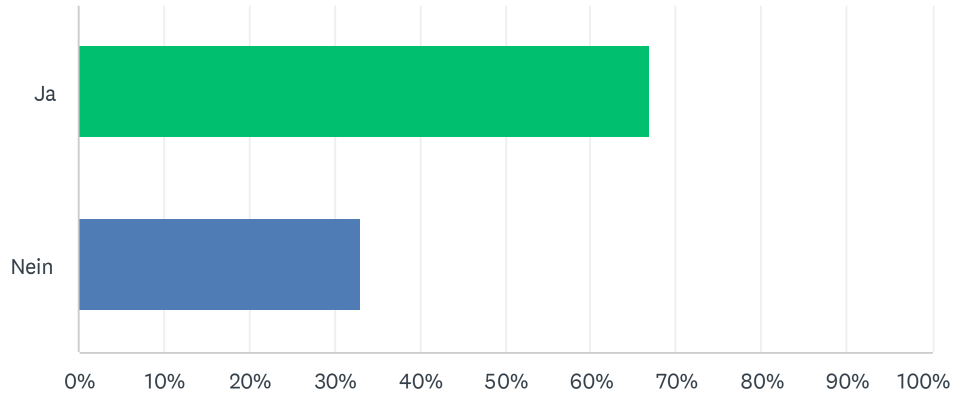

ANTWORTOPTIONEN	BEANTWORTUNGEN	
Ja	66.95%	79
Nein	33.05%	39
GESAMT		118

F44 Wie beurteilen Sie die an Ihrer Klinik vorhandene Weiterbildung?

Beantwortet: 118   Übersprungen: 4

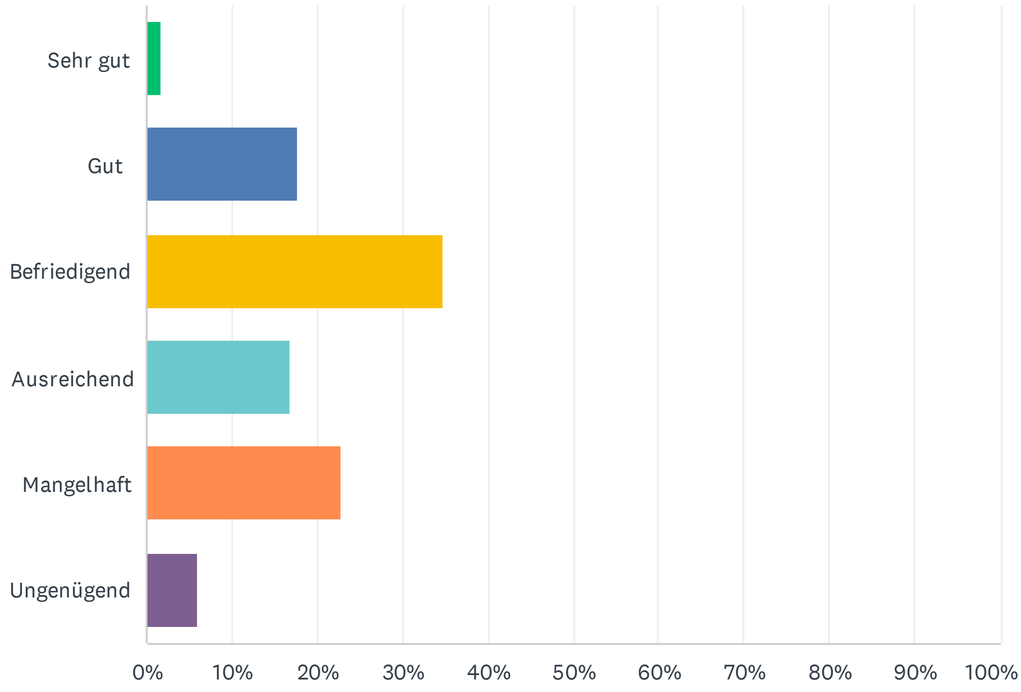

ANTWORTOPTIONEN	BEANTWORTUNGEN	
Sehr gut	1.69%	2
Gut	17.80%	21
Befriedigend	34.75%	41
Ausreichend	16.95%	20
Mangelhaft	22.88%	27
Ungenügend	5.93%	7
GESAMT		118

F45 Gibt es einen strukturierten Weiterbildungsplan?

Beantwortet: 118 Übersprungen: 4

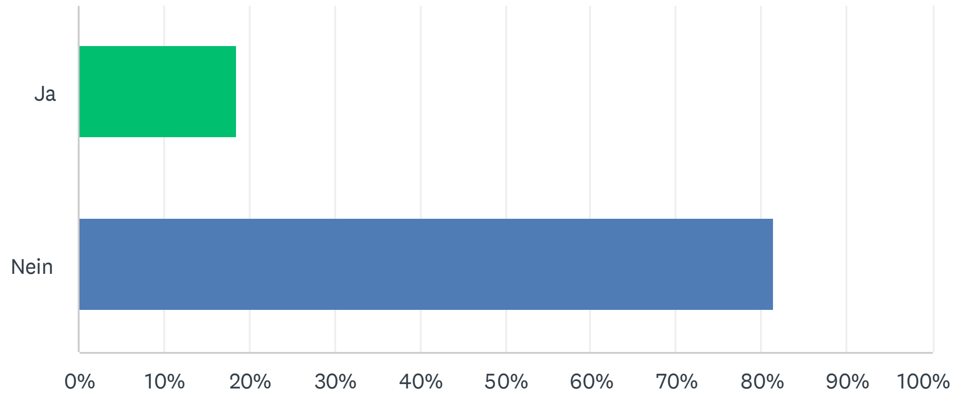

ANTWORTOPTIONEN	BEANTWORTUNGEN	
Ja	18.64%	22
Nein	81.36%	96
GESAMT		118

F46 Wird der strukturierte Weiterbildungsplan zuverlässig eingehalten?

Beantwortet: 38   Übersprungen: 84

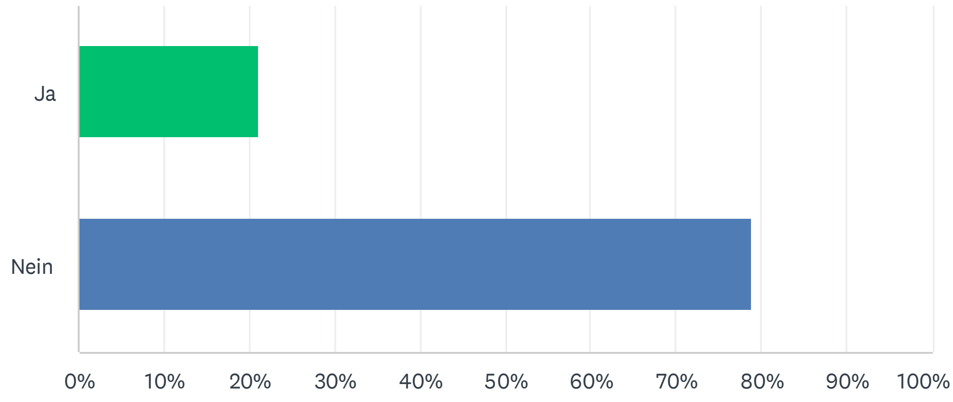

ANTWORTOPTIONEN	BEANTWORTUNGEN	
Ja	21.05%	8
Nein	78.95%	30
GESAMT		38

F47 Haben Sie den Eindruck, dass Sie die Fähigkeiten, die Sie während Ihrer bisher absolvierten Weiterbildungszeit erlernt haben, in gleicher Weise auch in kürzerer Zeit oder sogar effektiver hätten erlernen können?

Beantwortet: 118 Übersprungen: 4

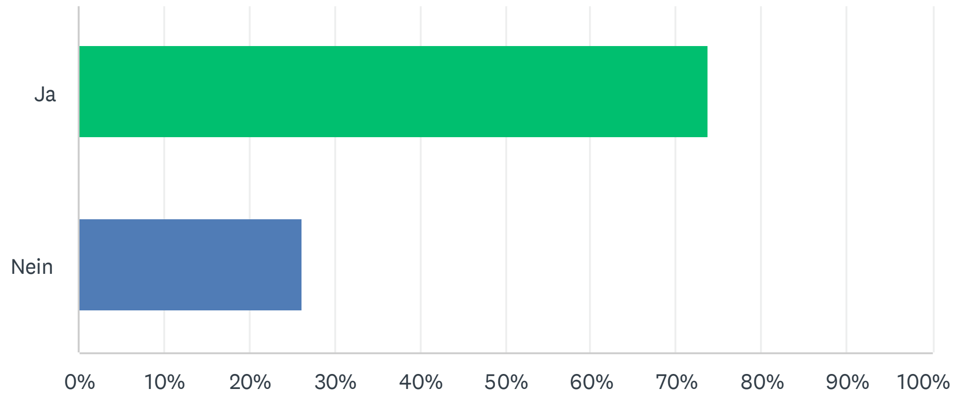

ANTWORTOPTIONEN	BEANTWORTUNGEN	
Ja	73.73%	87
Nein	26.27%	31
GESAMT		118

F48 Sind externe Fortbildungen notwendig, um den Facharztstandard bis zum Ende der Weiterbildung zu erlangen?

Beantwortet: 118   Übersprungen: 4

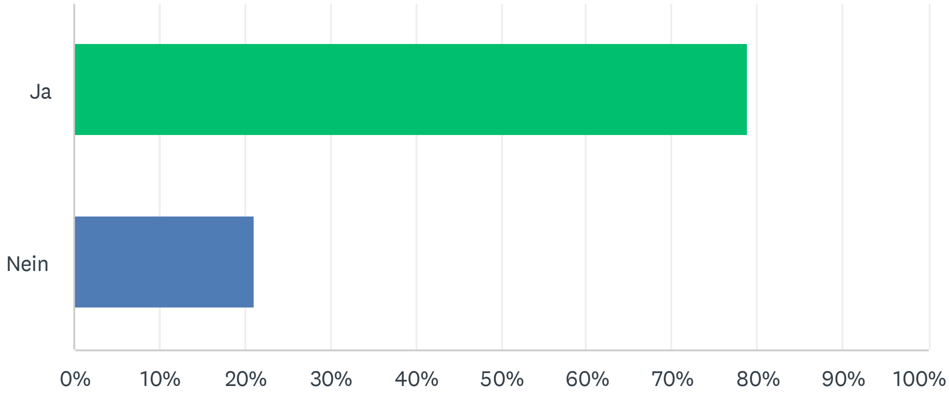

ANTWORTOPTIONEN	BEANTWORTUNGEN	
Ja	78.81%	93
Nein	21.19%	25
GESAMT		118

F49 Erhalten Sie finanzielle Zuschüsse zu externen Fortbildungen?

Beantwortet: 118   Übersprungen: 4

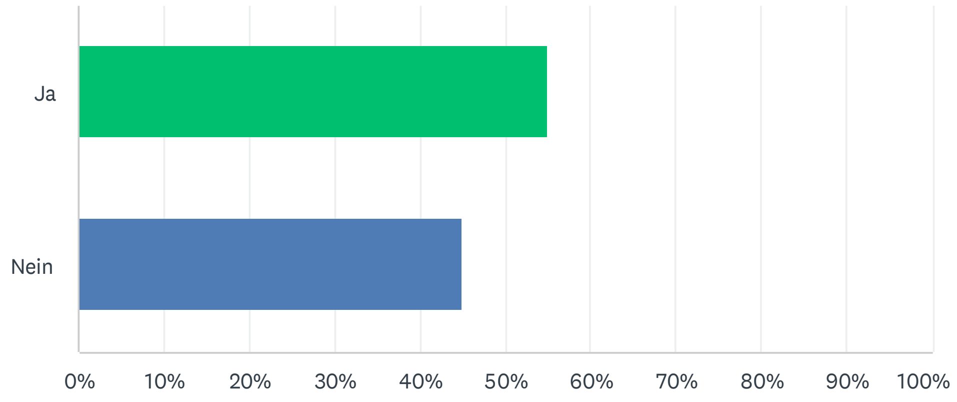

ANTWORTOPTIONEN	BEANTWORTUNGEN	
Ja	55.08%	65
Nein	44.92%	53
GESAMT		118

F50 Gibt es ein Oberarzt-Mentoren-Programm1 an Ihrer Klinik?1 Ein Oberarzt betreut junge Kollegen (max. 2-3) und ist erster Ansprechpartner beispielsweise in Fragen der Ausbildung.

Beantwortet: 118 Übersprungen: 4

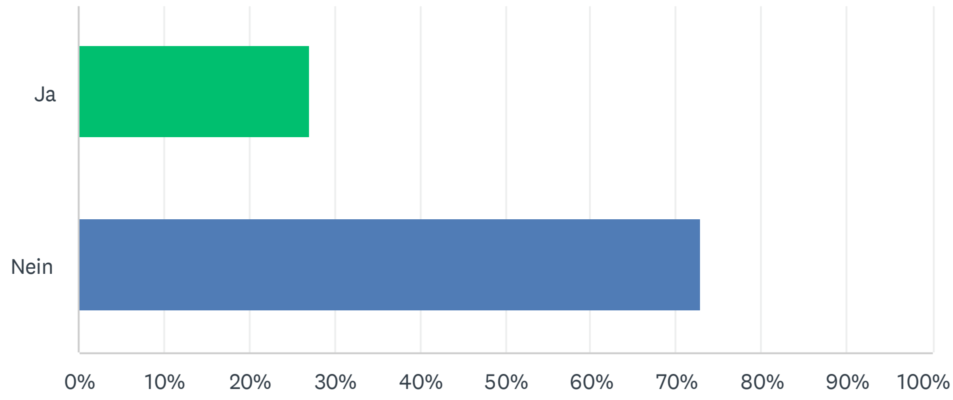

ANTWORTOPTIONEN	BEANTWORTUNGEN	
Ja	27.12%	32
Nein	72.88%	86
GESAMT		118

F51 Finden regelmäßige Weiterbildungsgespräche, wie gefordert, ein Mal jährlich statt?

Beantwortet: 118 Übersprungen: 4

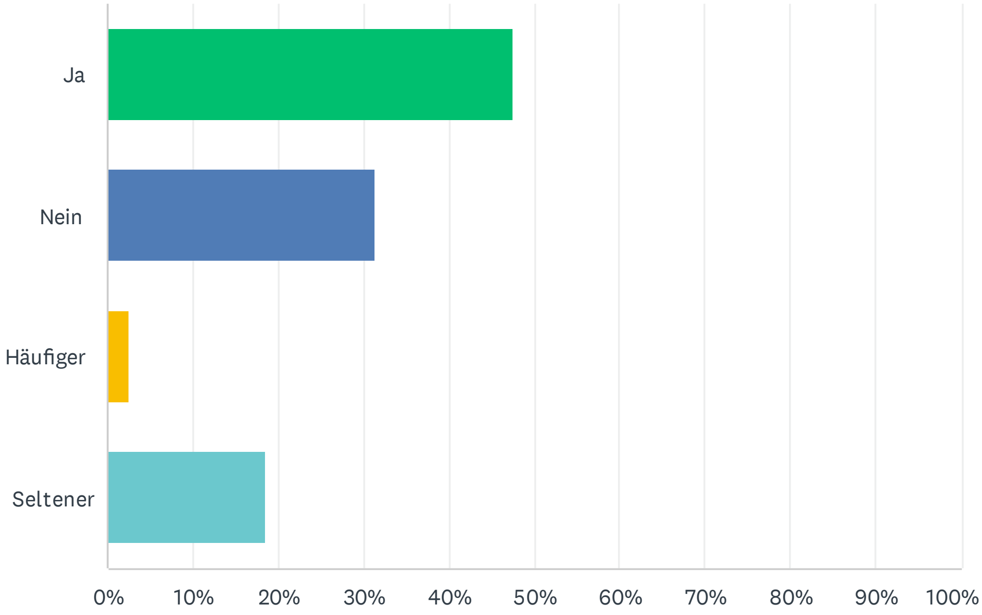

ANTWORTOPTIONEN	BEANTWORTUNGEN	
Ja	47.46%	56
Nein	31.36%	37
Häufiger	2.54%	3
Seltener	18.64%	22
GESAMT		118

F52 Empfinden Sie die Weiterbildungsgespräche als konstruktiv?

Beantwortet: 115 Übersprungen: 7

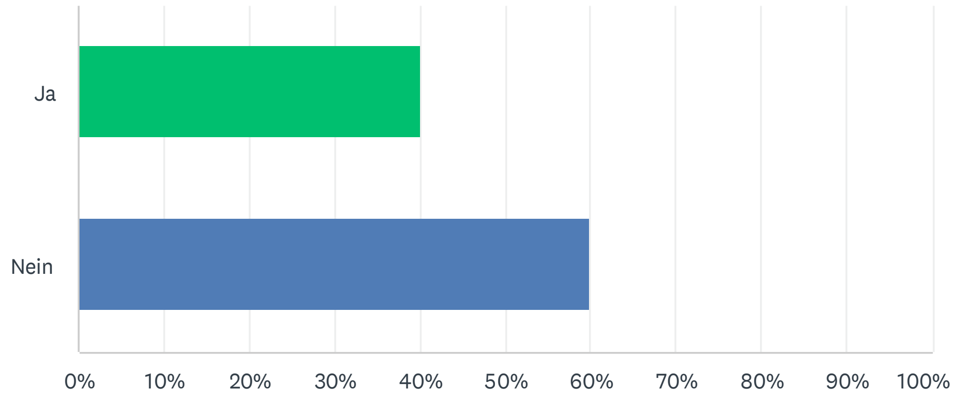

ANTWORTOPTIONEN	BEANTWORTUNGEN	
Ja	40.00%	46
Nein	60.00%	69
GESAMT		115

F53 Werden Studenten gegenüber Ärzten in Weiterbildung in Ihrer Klinik im OP bevorzugt als 1. Assistenz eingesetzt?

Beantwortet: 118 Übersprungen: 4

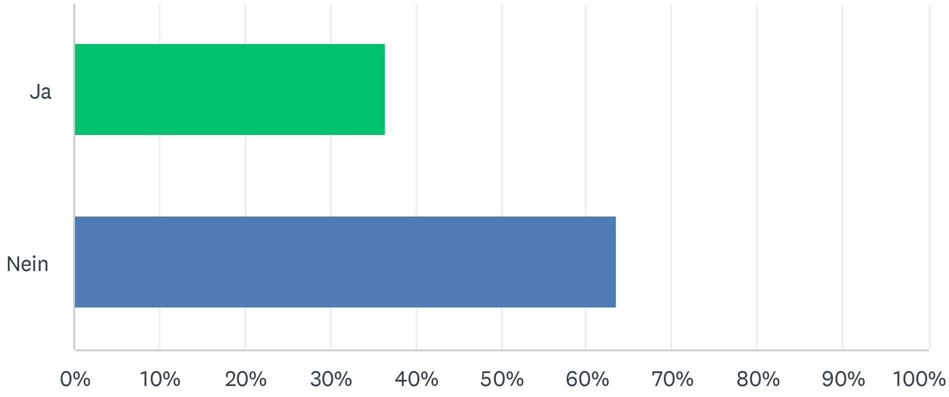

ANTWORTOPTIONEN	BEANTWORTUNGEN	
Ja	36.44%	43
Nein	63.56%	75
GESAMT		118

F54 Tendieren Sie eher zu einer Work-Life-Balance1 oder zu einer Work-Life-Separation2?1 Work-Life-Balance: Flexibler bzw. fließender Übergang zwischen Privat- und Berufsleben2 Work-Life-Separation: Klare Trennung zwischen Privat- und Berufsleben

Beantwortet: 118 Übersprungen: 4

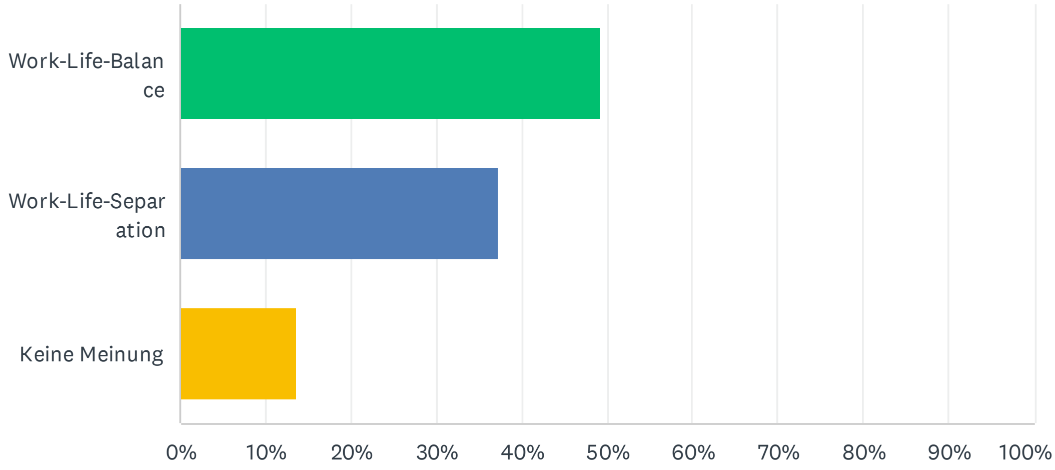

ANTWORTOPTIONEN	BEANTWORTUNGEN	
Work-Life-Balance	49.15%	58
Work-Life-Separation	37.29%	44
Keine Meinung	13.56%	16
GESAMT		118

F55 Welchen Stellenwert nimmt Ihre Gesundheit für Sie gegenüber dem Berufsalltag ein?

Beantwortet: 117 Übersprungen: 5

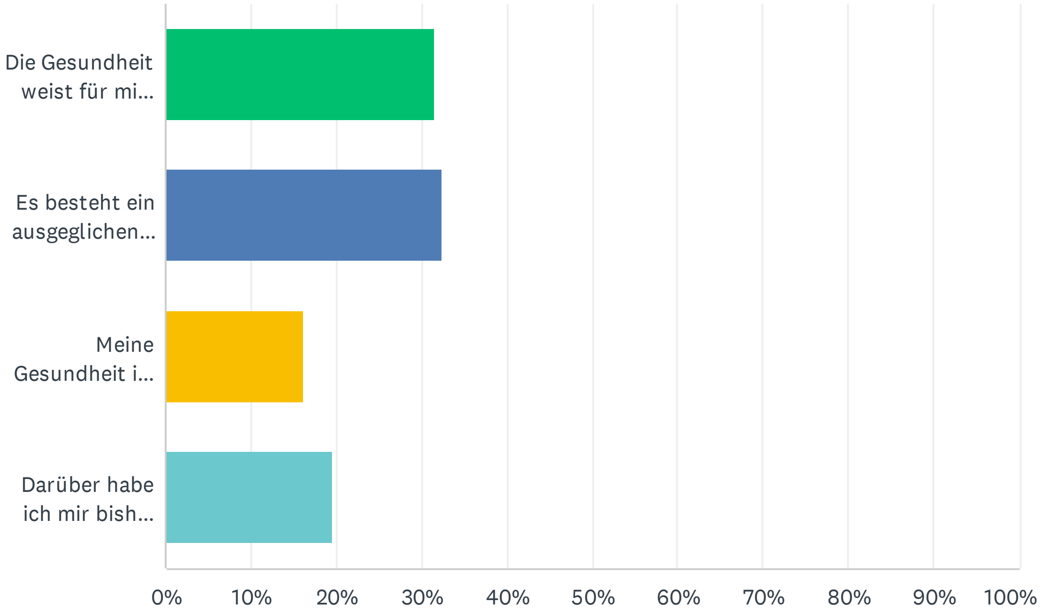

ANTWORTOPTIONEN	BEANTWORTUNGEN	
Die Gesundheit weist für mich gegenüber des Berufs nach dem Grundsatz „Gesunde Ärzte für gesunde Patienten" eine übergeordnete Bedeutung auf	31.62%	37
Es besteht ein ausgeglichenes Verhältnis zwischen meiner Gesundheit und meinem Beruf	32.48%	38
Meine Gesundheit ist für mich von untergeordneter Bedeutung	16.24%	19
Darüber habe ich mir bisher noch keine Gedanken gemacht	19.66%	23
GESAMT		117

F56 Haben Sie den Eindruck, dass die „Hemmschwelle“, sich krank zu melden, unter der zunehmenden Arbeitsbelastung sinkt?

Beantwortet: 118   Übersprungen: 4

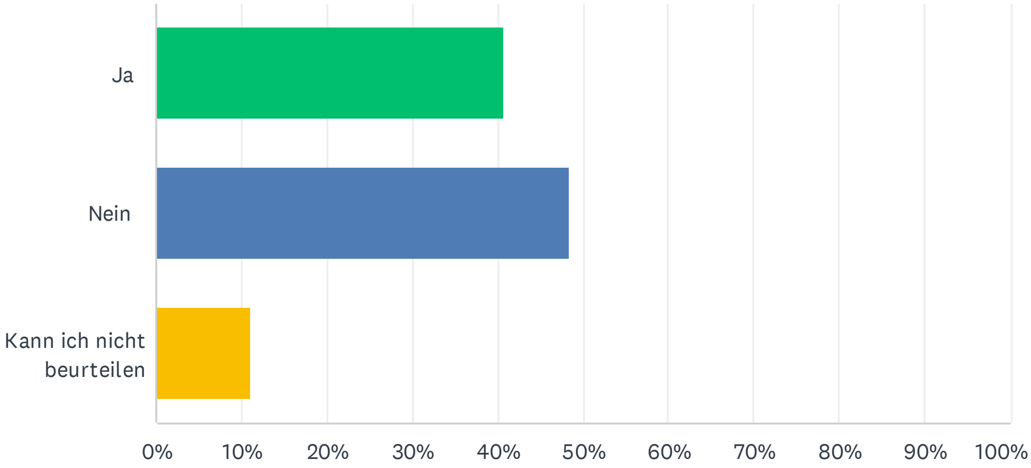

ANTWORTOPTIONEN	BEANTWORTUNGEN	
Ja	40.68%	48
Nein	48.31%	57
Kann ich nicht beurteilen	11.02%	13
GESAMT		118

F57 Wie viele Krankheitstage hatten Sie im vergangenen Jahr?

Beantwortet: 116 Übersprungen: 6

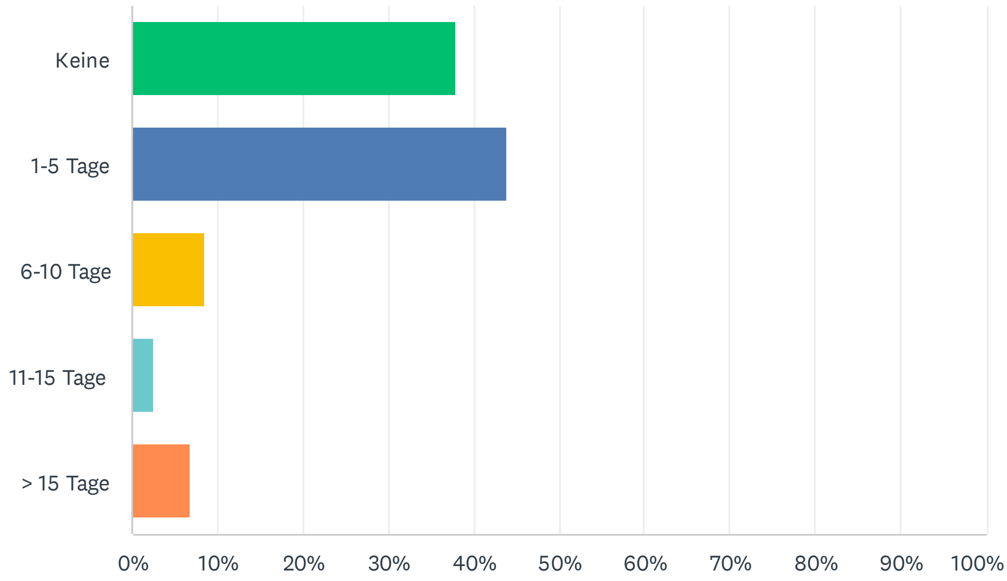

ANTWORTOPTIONEN	BEANTWORTUNGEN	
Keine	37.93%	44
1-5 Tage	43.97%	51
6-10 Tage	8.62%	10
11-15 Tage	2.59%	3
> 15 Tage	6.90%	8
GESAMT		116

F58 Hatten Sie das Gefühl, dass die Arbeitsbelastung Grund für Ihren Arbeitsausfall war?

Beantwortet: 116 Übersprungen: 6

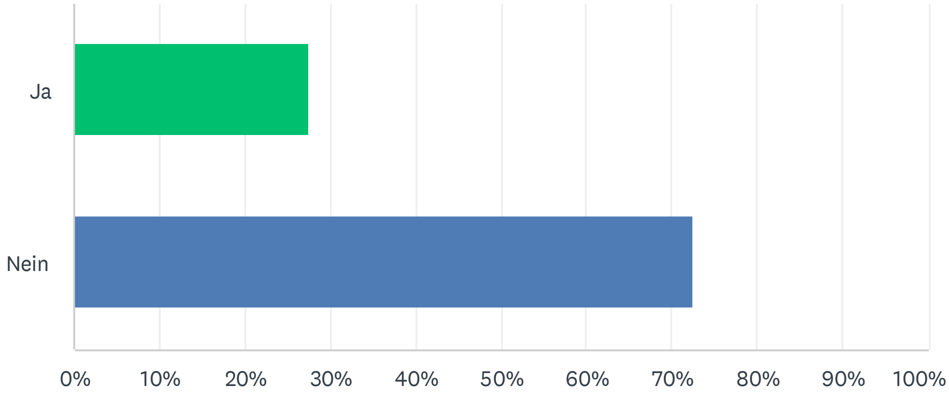

ANTWORTOPTIONEN	BEANTWORTUNGEN	
Ja	27.59%	32
Nein	72.41%	84
GESAMT		116

F59 Haben Sie schon einmal Medikamente eingenommen, um trotz Krankheit zur Arbeit erscheinen zu können?

Beantwortet: 118 Übersprungen: 4

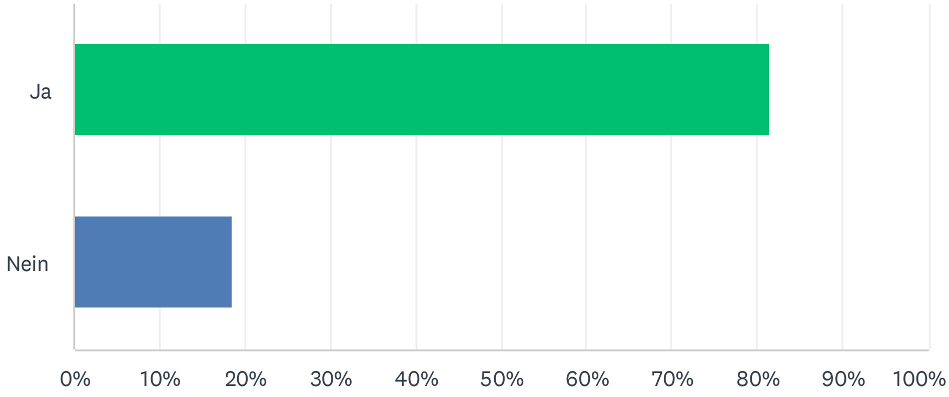

ANTWORTOPTIONEN	BEANTWORTUNGEN	
Ja	81.36%	96
Nein	18.64%	22
GESAMT		118

F60 Wenn Sie in Ihrer jetzigen Situation die Wahl zwischen mehr Einkommen bei entsprechend weniger freier Zeit oder mehr freier Zeit bei entsprechend weniger Einkommen hätten, wie würden Sie sich entscheiden?

Beantwortet: 118 Übersprungen: 4

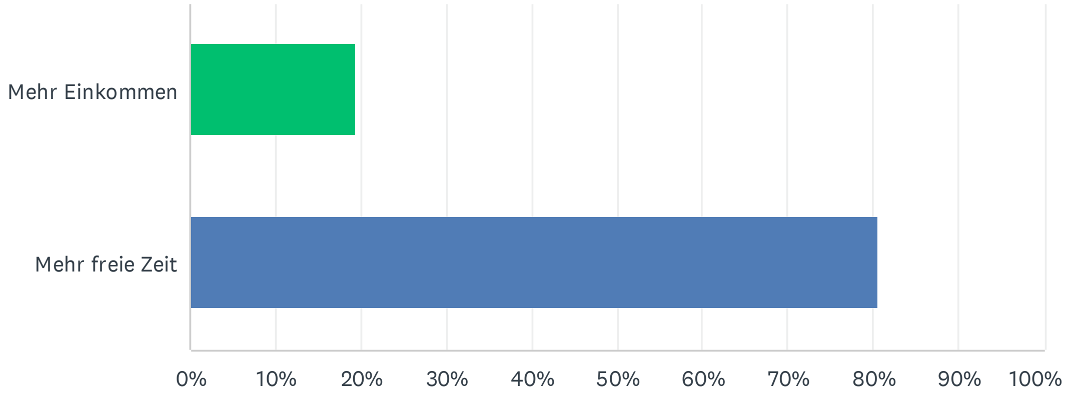

ANTWORTOPTIONEN	BEANTWORTUNGEN	
Mehr Einkommen	19.49%	23
Mehr freie Zeit	80.51%	95
GESAMT		118

F61 Präferieren Sie die Auszahlung geleisteter Überstunden oder die Vergütung der Überstunden als Freizeitausgleich?

Beantwortet: 118 Übersprungen: 4

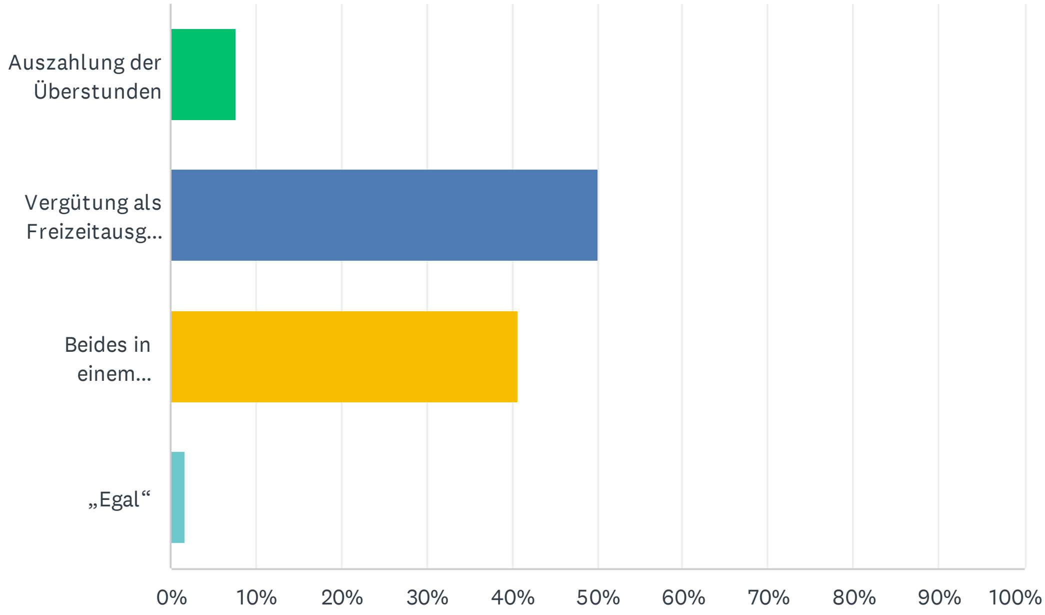

ANTWORTOPTIONEN	BEANTWORTUNGEN	
Auszahlung der Überstunden	7.63%	9
Vergütung als Freizeitausgleich	50.00%	59
Beides in einem ausgewogenen Verhältnis zueinander	40.68%	48
„Egal"	1.69%	2
GESAMT		118

F62 Beeinträchtigt Ihre derzeitige Arbeitsbelastung Ihr Privatleben überwiegend negativ?

Beantwortet: 117 Übersprungen: 5

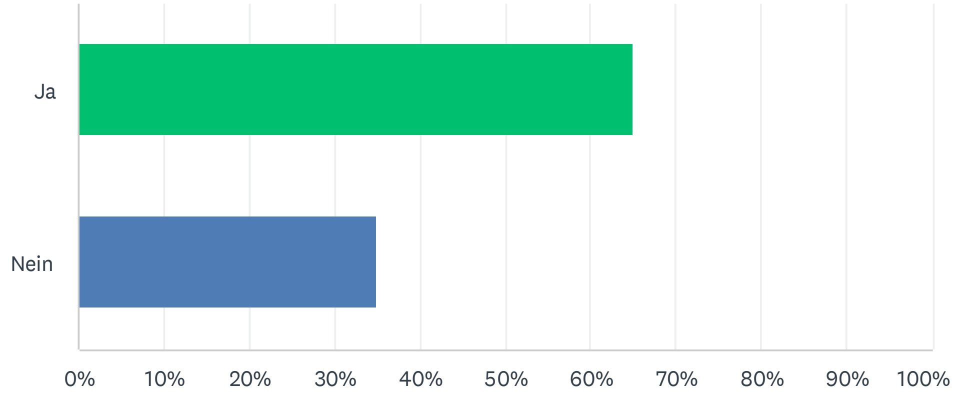

ANTWORTOPTIONEN	BEANTWORTUNGEN	
Ja	64.96%	76
Nein	35.04%	41
GESAMT		117

F63 Beurteilen Sie die derzeitigen Arbeitsbedingungen in Ihrer Klinik als familienfreundlich?

Beantwortet: 117   Übersprungen: 5

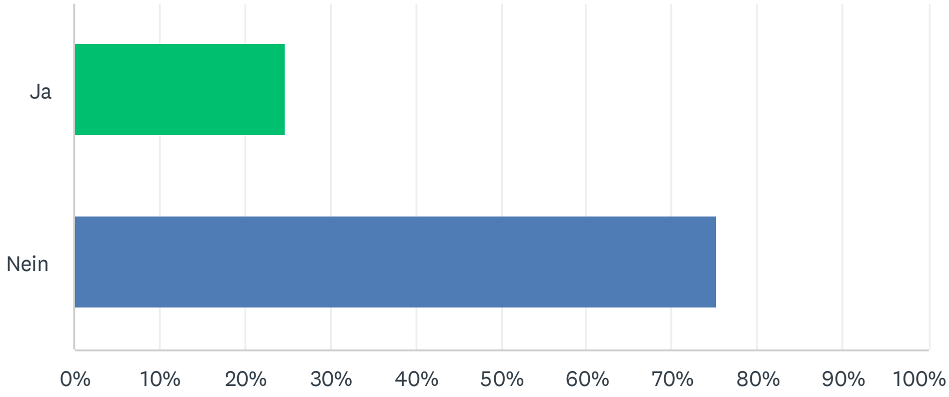

ANTWORTOPTIONEN	BEANTWORTUNGEN	
Ja	24.79%	29
Nein	75.21%	88
GESAMT		117

F64 Stellt für Sie der Wechsel in die Teilzeit, unabhängig von Kindern bzw. Familie, eine alternative Option dar?

Beantwortet: 118   Übersprungen: 4

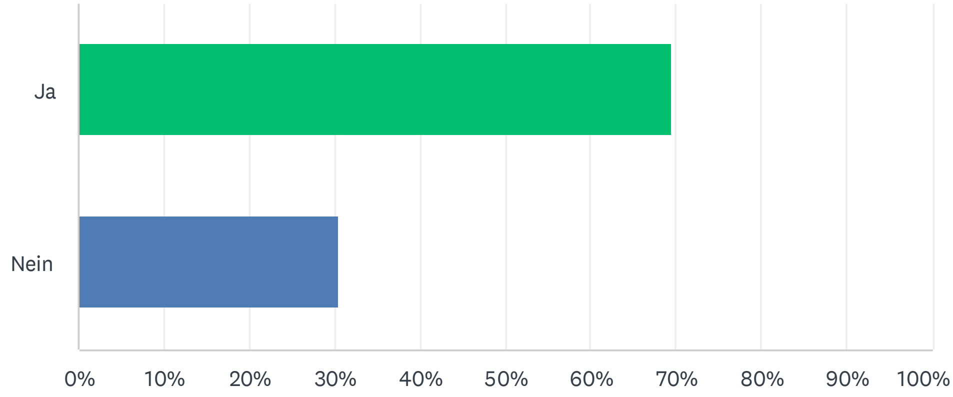

ANTWORTOPTIONEN	BEANTWORTUNGEN	
Ja	69.49%	82
Nein	30.51%	36
GESAMT		118

F65 Erhalten Sie ausreichend Möglichkeiten, die Lehre der Studenten mitzugestalten?

Beantwortet: 117   Übersprungen: 5

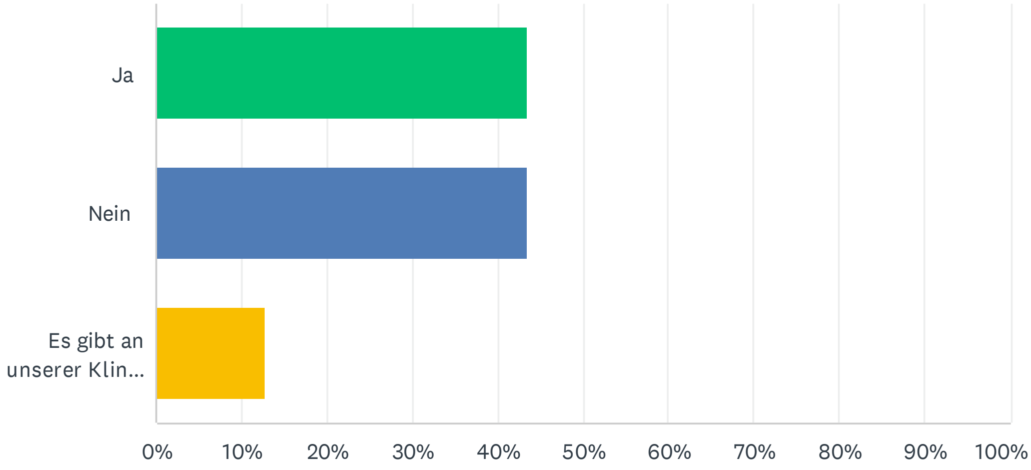

ANTWORTOPTIONEN	BEANTWORTUNGEN	
Ja	43.59%	51
Nein	43.59%	51
Es gibt an unserer Klinik keine Lehre	12.82%	15
GESAMT		117

F66 Haben Sie schon einmal von der aktuellen DGGG-Nachwuchskampagne „GYN-WERDEN“ gehört?

Beantwortet: 117 Übersprungen: 5

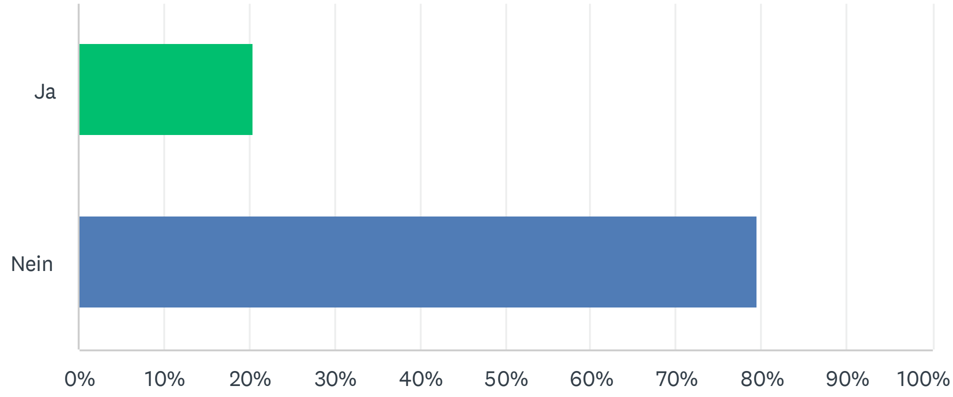

ANTWORTOPTIONEN	BEANTWORTUNGEN	
Ja	20.51%	24
Nein	79.49%	93
GESAMT		117

F67 Gibt es an Ihrer Klinik Angebote wie beispielweise die „DGGG-Summer-School“, um Nachwuchs zu rekrutieren?

Beantwortet: 117 Übersprungen: 5

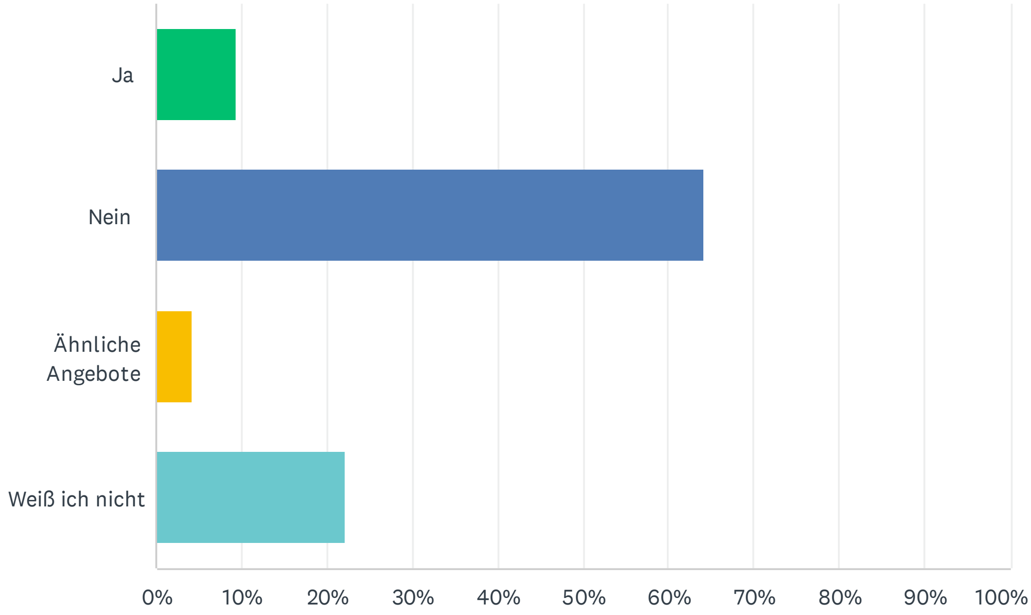

ANTWORTOPTIONEN	BEANTWORTUNGEN	
Ja	9.40%	11
Nein	64.10%	75
Ähnliche Angebote	4.27%	5
Weiß ich nicht	22.22%	26
GESAMT		117

F68 Fördert Ihre Klinik Angebote, um Sie an Ihrer jetzigen Klinik zu halten?

Beantwortet: 117 Übersprungen: 5

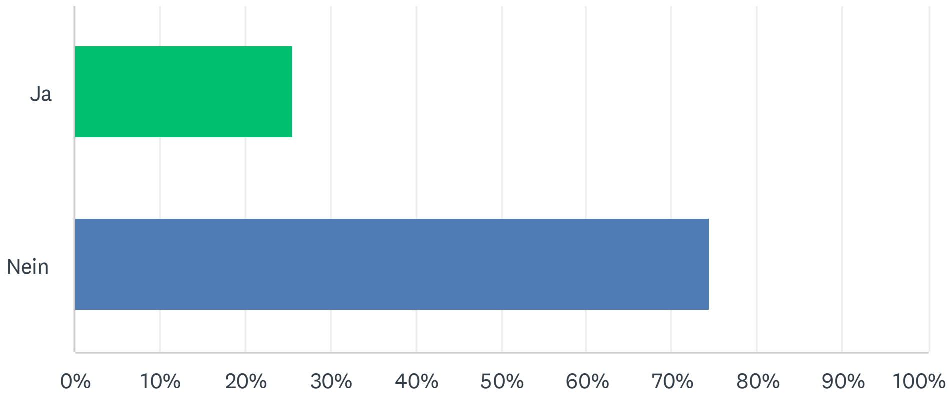

ANTWORTOPTIONEN	BEANTWORTUNGEN	
Ja	25.64%	30
Nein	74.36%	87
GESAMT		117

F69 Welche Angebote bietet Ihre Klinik an, damit Sie weiterhin für diese arbeiten?

Beantwortet: 30   Übersprungen: 92

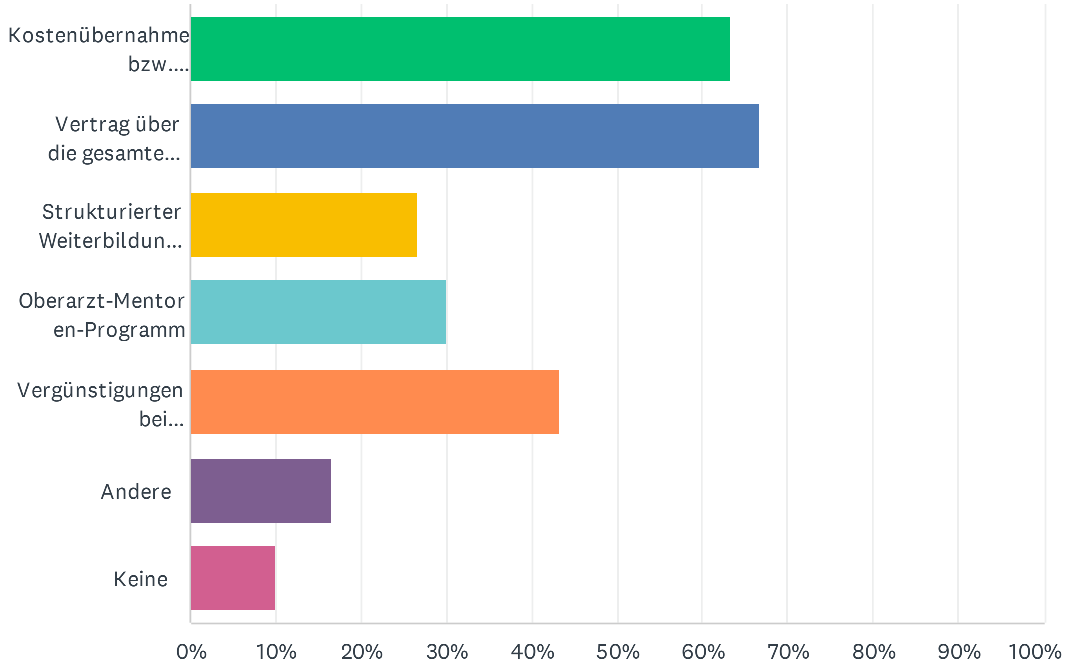

ANTWORTOPTIONEN	BEANTWORTUNGEN	
Kostenübernahme bzw. Kostenzuschuss für Fortbildungen	63.33%	19
Vertrag über die gesamte Weiterbildungszeit	66.67%	20
Strukturierter Weiterbildungsplan (Rotationsplan)	26.67%	8
Oberarzt-Mentoren-Programm	30.00%	9
Vergünstigungen bei Drittanbietern (Fitnessstudio, Mobilfunkvertrag, Fahrradreparatur, …)	43.33%	13
Andere	16.67%	5
Keine	10.00%	3
Befragte insgesamt: 30		

F70 Wünschen Sie sich, abseits Ihrer alltäglichen Arbeit, durch die Klinik Angebote in Ihrer freien Zeit (z.B. Sportangebote, Fitnessstudio, Mobilfunkvertrag, Fahrradreparatur, Vergünstigungen, Sonderkonditionen oder Rabattangebote, …)?

Beantwortet: 116 Übersprungen: 6

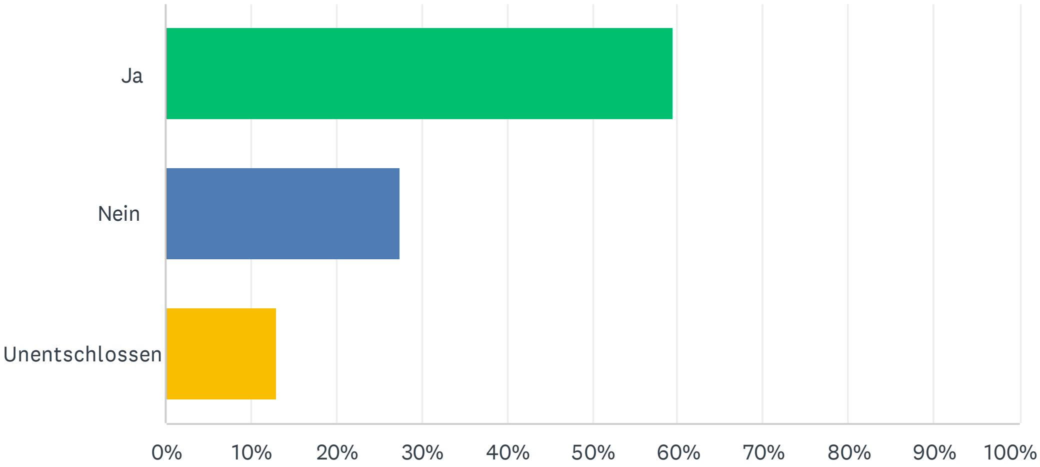

ANTWORTOPTIONEN	BEANTWORTUNGEN	
Ja	59.48%	69
Nein	27.59%	32
Unentschlossen	12.93%	15
GESAMT		116

F71 Welche Gründe haben Sie dazu bewegt, sich an Ihrer jetzigen Klinik zu bewerben (Mehrfachauswahl)?

Beantwortet: 116 Übersprungen: 6

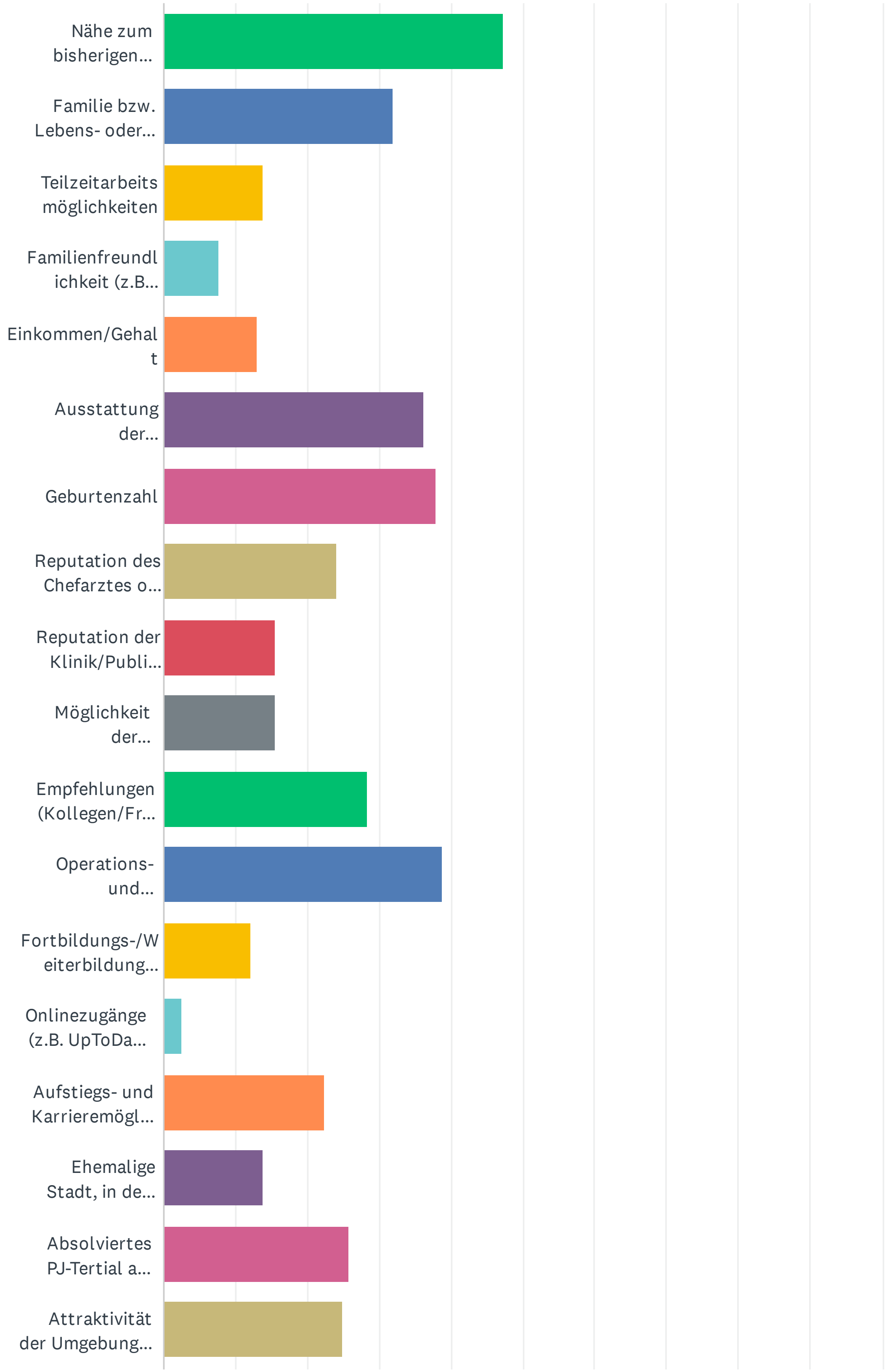

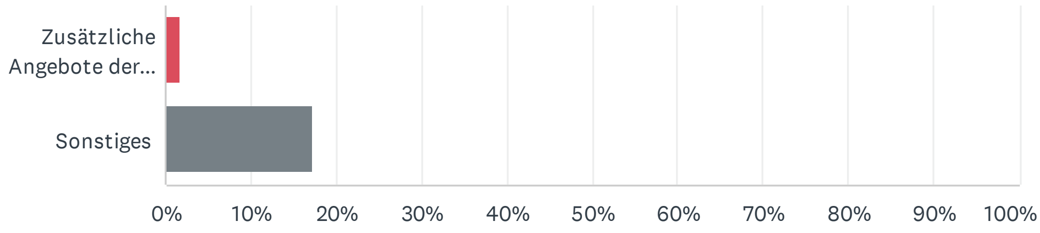

ANTWORTOPTIONEN	BEANTWORTUNGEN	
Nähe zum bisherigen Wohnort	47.41%	55
Familie bzw. Lebens- oder Ehepartner	31.90%	37
Teilzeitarbeitsmöglichkeiten	13.79%	16
Familienfreundlichkeit (z.B. Kita-Platz)	7.76%	9
Einkommen/Gehalt	12.93%	15
Ausstattung der Klinik/Abteilung	36.21%	42
Geburtenzahl	37.93%	44
Reputation des Chefarztes oder anderer Oberärzte	24.14%	28
Reputation der Klinik/Publikationen	15.52%	18
Möglichkeit der wissenschaftlichen Tätigkeit	15.52%	18
Empfehlungen (Kollegen/Freunde)	28.45%	33
Operations- und Leistungsspektrum	38.79%	45
Fortbildungs-/Weiterbildungsangebote	12.07%	14
Onlinezugänge (z.B. UpToDate, Thieme eRef, Springer, …)	2.59%	3
Aufstiegs- und Karrieremöglichkeiten	22.41%	26
Ehemalige Stadt, in der man sein Studium absolviert hat	13.79%	16
Absolviertes PJ-Tertial an der Klinik/Abteilung	25.86%	30
Attraktivität der Umgebung (Freizeitangebote, Kultur, …)	25.00%	29
Zusätzliche Angebote der Klinik	1.72%	2
Sonstiges	17.24%	20
Befragte insgesamt: 116		

F72 Wären Sie dazu bereit, bei anhaltender Unzufriedenheit Ihre Klinik zu wechseln?

Beantwortet: 116 Übersprungen: 6

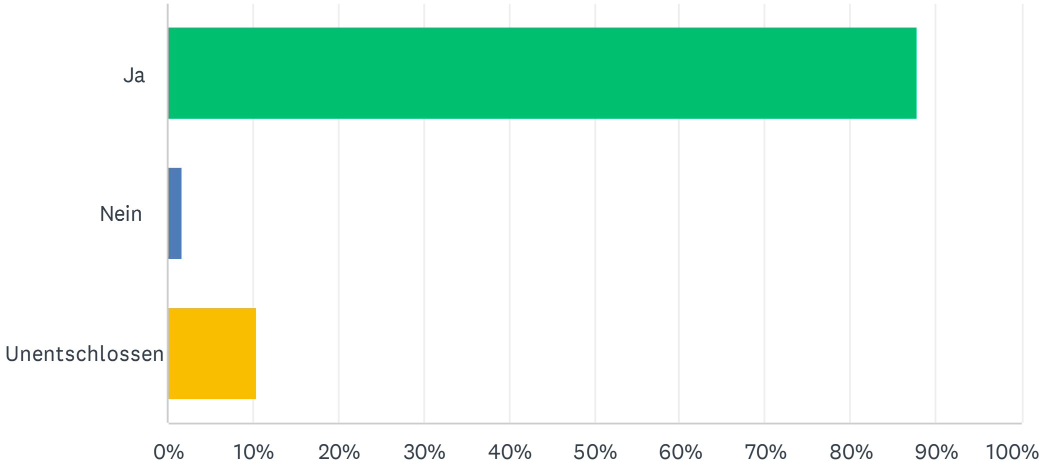

ANTWORTOPTIONEN	BEANTWORTUNGEN	
Ja	87.93%	102
Nein	1.72%	2
Unentschlossen	10.34%	12
GESAMT		116

F73 Erinnern Sie sich konkret an eine oder mehrere Situation, in der die Patientenversorgung gefährdet war?

Beantwortet: 116 Übersprungen: 6

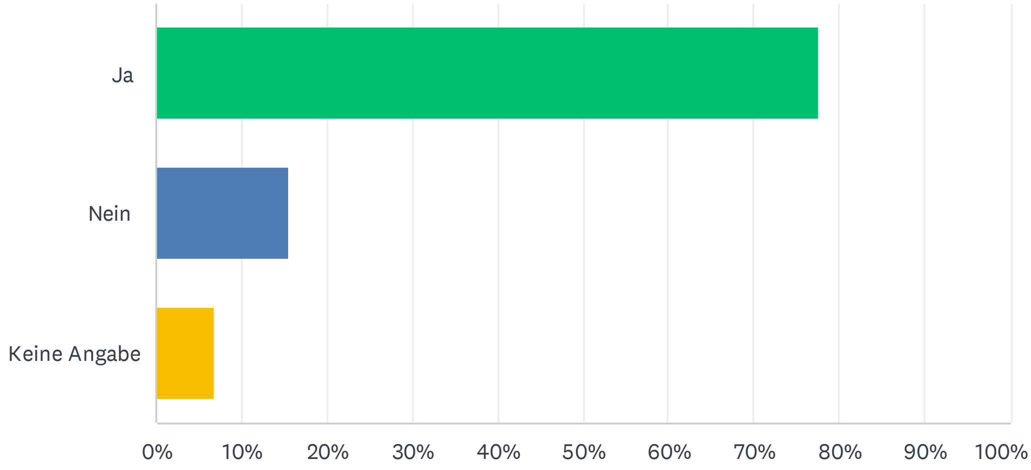

ANTWORTOPTIONEN	BEANTWORTUNGEN	
Ja	77.59%	90
Nein	15.52%	18
Keine Angabe	6.90%	8
GESAMT		116

F74 Nennen Sie einen oder mehrere Gründe, welcher bzw. welche aus Ihrer Sicht maßgeblich zur Gefährdung der Patientenversorgung beigetragen haben.

Beantwortet: 90   Übersprungen: 32

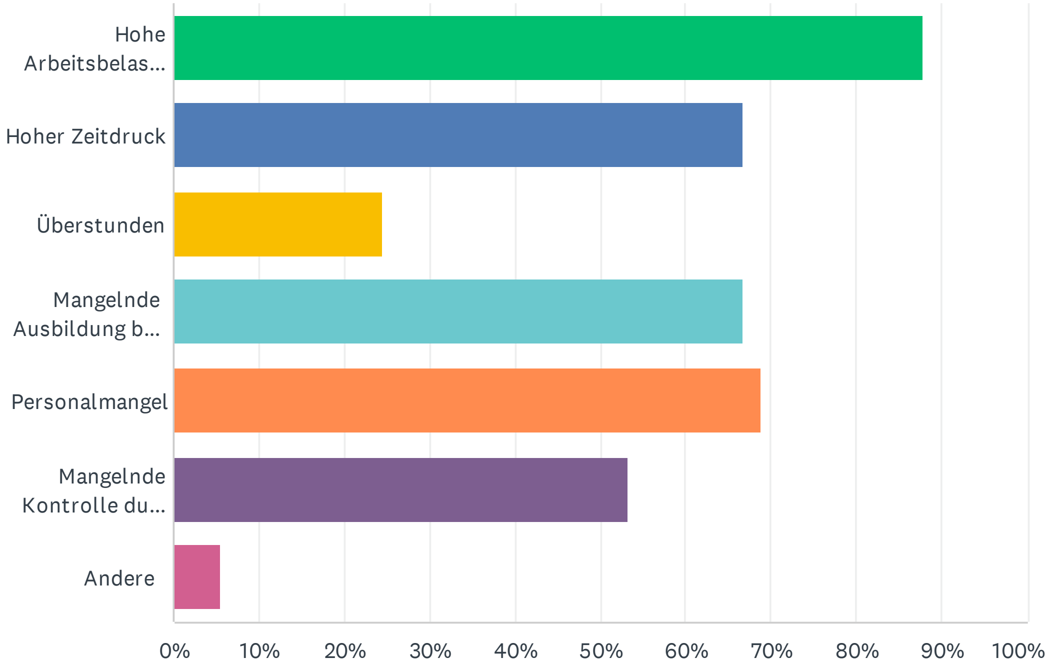

ANTWORTOPTIONEN	BEANTWORTUNGEN	
Hohe Arbeitsbelastung	87.78%	79
Hoher Zeitdruck	66.67%	60
Überstunden	24.44%	22
Mangelnde Ausbildung bzw. Erfahrung	66.67%	60
Personalmangel	68.89%	62
Mangelnde Kontrolle durch erfahrene Oberärzte	53.33%	48
Andere	5.56%	5
Befragte insgesamt: 90		

F75 Wer kodiert in Ihrer Klinik Diagnosen und Prozeduren?

Beantwortet: 116 Übersprungen: 6

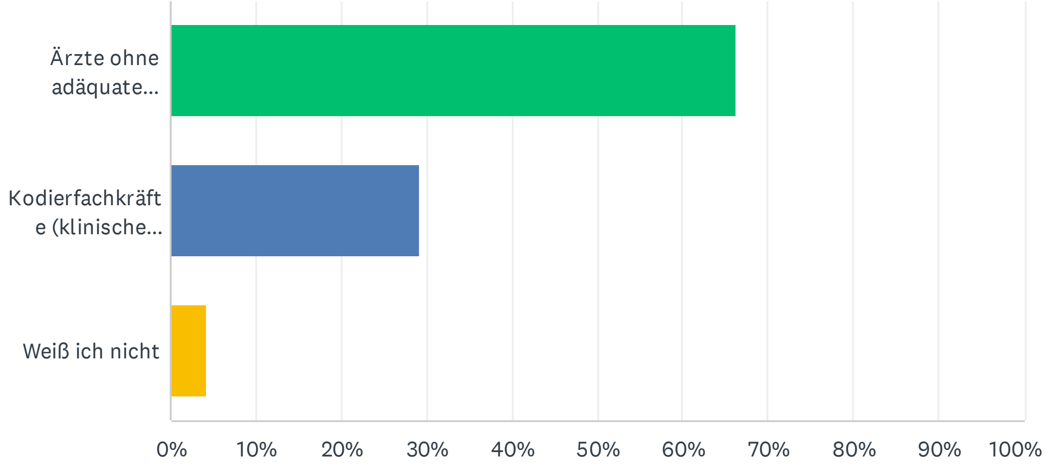

ANTWORTOPTIONEN	BEANTWORTUNGEN	
Ärzte ohne adäquate Kodierkenntnisse bzw. Schulung	66.38%	77
Kodierfachkräfte (klinische Kodierer) bzw. DRG-Beauftragte mit entsprechenden Kenntnissen	29.31%	34
Weiß ich nicht	4.31%	5
GESAMT		116

F76 Wussten Sie bei Ihrer Einstellung, was die Opt-Out-Regelung beinhaltet?

Beantwortet: 116 Übersprungen: 6

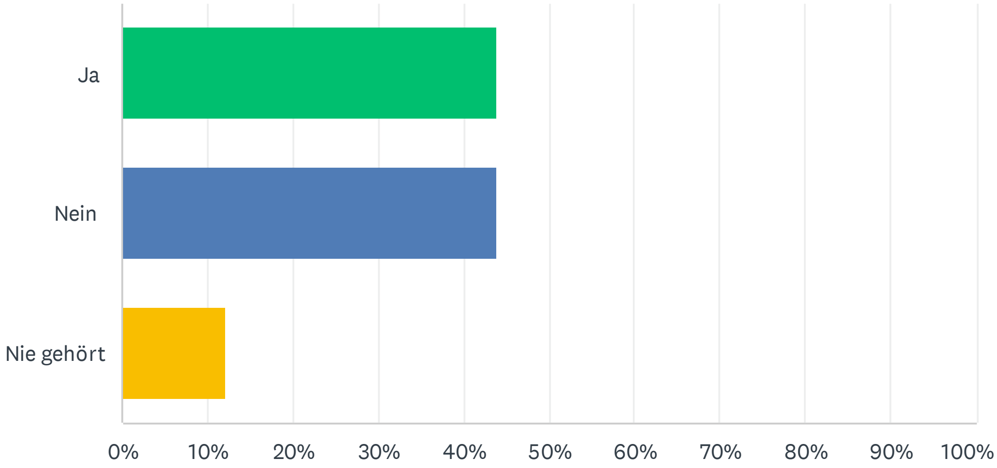

ANTWORTOPTIONEN	BEANTWORTUNGEN	
Ja	43.97%	51
Nein	43.97%	51
Nie gehört	12.07%	14
GESAMT		116

F77 Haben Sie die Opt-Out-Regelung unterschrieben?

Beantwortet: 115 Übersprungen: 7

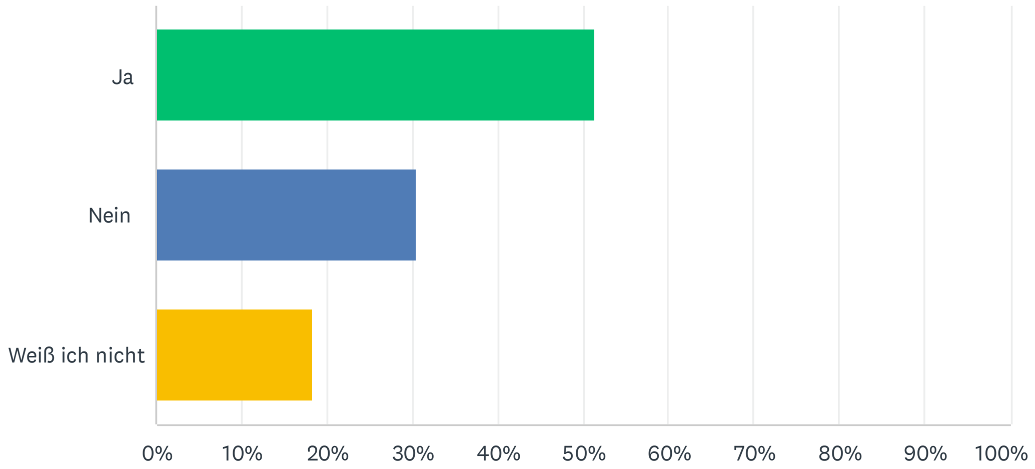

ANTWORTOPTIONEN	BEANTWORTUNGEN	
Ja	51.30%	59
Nein	30.43%	35
Weiß ich nicht	18.26%	21
GESAMT		115

F78 War die Opt-Out-Regelung Bedingung für Ihre Einstellung?

Beantwortet: 116 Übersprungen: 6

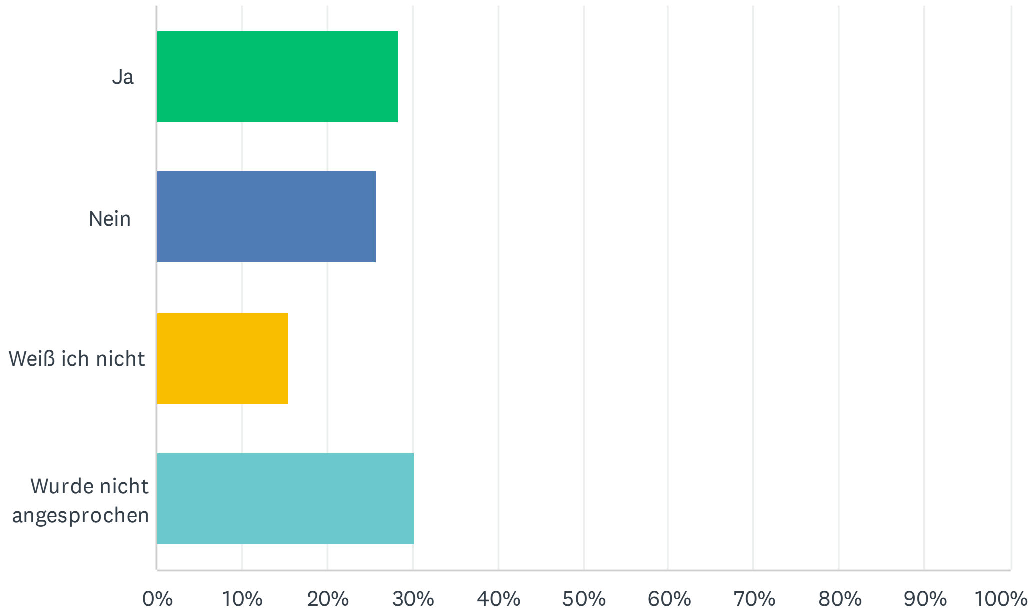

ANTWORTOPTIONEN	BEANTWORTUNGEN	
Ja	28.45%	33
Nein	25.86%	30
Weiß ich nicht	15.52%	18
Wurde nicht angesprochen	30.17%	35
GESAMT		116

F79 Gibt es an Ihrer Klinik Angebote, über belastende Erlebnisse im Rahmen einer Supervision zu sprechen?

Beantwortet: 116 Übersprungen: 6

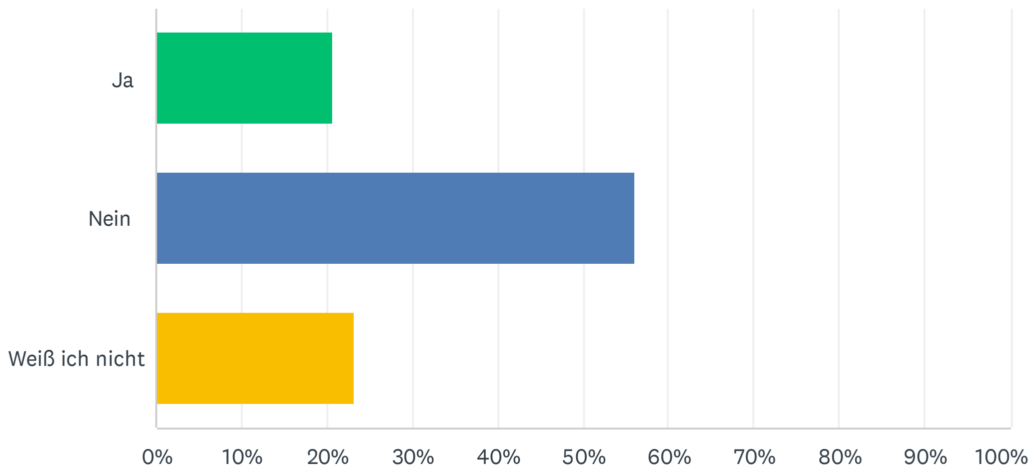

ANTWORTOPTIONEN	BEANTWORTUNGEN	
Ja	20.69%	24
Nein	56.03%	65
Weiß ich nicht	23.28%	27
GESAMT		116

F80 Nennen Sie, mit der höchsten Relevanz beginnend, die Ihrer Meinung nach drei größten Probleme an Ihrer Klinik.

Beantwortet: 101 Übersprungen: 21

ANTWORTOPTIONEN	BEANTWORTUNGEN	
1.)	100.00%	101
2.)	97.03%	98
3.)	90.10%	91

F81 Nennen Sie drei effektive Lösungsansätze, welche die aktuelle Situation aus Ihrer Sicht verbessern könnten.

Beantwortet: 94 Übersprungen: 28

ANTWORTOPTIONEN	BEANTWORTUNGEN	
1.)	100.00%	94
2.)	94.68%	89
3.)	82.98%	78
